# Supplementary material for: Meta-analysis models relaxing the random-effects normality assumption: methodological systematic review and simulation study
Source: BMC Med Res Methodol. 2025 Oct 16;25:231. doi: 10.1186/s12874-025-02658-3 (PMC12532406; doi:10.1186/s12874-025-02658-3)
Supplement: Supplementary file 1 — Supplementary Material 1: Supplementary Table 1. Characteristics of all the eligible articles identified through the systematic review Supplementary Table 2. Bayesian models and scenarios included (✔) or excluded (✘) from the simulation study due to lack of convergence in more than 5% of the datasets Supplementary Table 3. Summary of performance metrics for the common-mean mixture model, reporting results separately for outlying and non-outlying studies. The metrics include mean absolute bias, percent relative bias, mean squared error (MSE), and normalized MSE, for both standard deviation components of the model Supplementary Table 4. Mean absolute bias of the estimated skewness coefficient ($\:\gamma\:$) from the skew-normal model in scenarios where data were generated from a skew-normal distribution Supplementary Table 5. Estimated means with credible intervals of the concentration parameter ($\:\alpha\:$) from the five DP models for the different simulation scenarios Supplementary Table 6. Mean and variance of the random-effects distribution estimates along with their 95% Confidence/Credible Intervals for each selected simulated dataset using all models evaluated in the simulation study. The 95% prediction intervals are also presented Supplementary Table 7. Cluster probabilities for each selected simulated dataset using the respective DP model. Supplementary Fig. 1 Flow chart of the article selection process. Supplementary Fig. 2. Density plot of the skew normal distribution with loca [file 12874_2025_2658_MOESM1_ESM.docx]

# Supplementary

**Supplementary Table 1**. Characteristics of all the eligible articles identified through the systematic review.

| **Title** | **First author** | **Journal** | **Publication**  **year** | **Suggested random-effects distribution(s)** | **Bayesian/**  **Frequentist framework** | **Software/**  **code available** |
| --- | --- | --- | --- | --- | --- | --- |
| A new approach to outliers in meta-analysis | Baker R | Health Care Management Science | 2007 | Beta distribution & skewed extensions of t-distribution | Frequentist & Bayesian | Code not provided |
| New models for describing outliers in meta-analysis | Baker R | Research Synthesis Methods | 2016 | Mixture of a normal distribution & lagged normal distribution | Frequentist | Code provided in Supplementary |
| Nonparametric Bayesian Approach to Treatment Ranking in Network Meta-Analysis with Application to Comparisons of Antidepressants | Barrientos A | Journal of the Royal Statistical Society Series C: Applied Statistics | 2024 | Dirichlet Process prior mixture | Bayesian | Implemented in the R package *CBnetworkMA*^1^ |
| A finite mixture method for outlier detection and robustness in meta-analysis | Beath K | Research Synthesis Methods | 2014 | Mixture of normal distributions including co-variates | Frequentist | Implemented in the  R package *metaplus*^2^ |
| Objective Bayesian Meta-Analysis Based on Generalized Marginal Multivariate Random Effects Model | Bodnar O | Bayesian analysis | 2024 | Multivariate t-distributions | Bayesian | Implemented in the  R-package *BayesMultMeta*^3^ |
| Nonparametric Estimation of Effect Heterogeneity in Rare Events Meta-Analysis: Bivariate, Discrete Mixture Model. | Böhning D | Lobachevskii Journal of Mathematics | 2021 | Mixture of Poisson regression model | Frequentist | Code provided in Supplementary |
| Bayesian non-parametric meta-analysis using Polya-tree mixture models | Branscum A | Biometrics | 2008 | Polya-tree prior mixture | Bayesian | Code not provided |
| Examining How Context Changes Intervention Impact: The Use of Effect Sizes in Multilevel Mixture Meta-Analysis | Brown CH | Child Development Perspectives | 2008 | Mixture of two normal distributions | Frequentist | Code provided in Supplementary |
| A Bayesian semi-parametric model for random effects meta-analysis | Burr D | Journal of American Statistical Association | 2011 | Dirichlet Process prior mixture | Bayesian | Implemented in the  R package *bspmma*^4^ |
| A meta-analysis of studies on the association of the platelet PlA polymorphism of glycoprotein IIIa and risk of coronary heart disease | Burr D | Statistics in Medicine | 2003 | Dirichlet Process prior mixture | Bayesian | Implemented in the  R package *bspmma*^4^ |
| A Bayesian nonparametric meta-analysis model for estimating the reference interval | Cao W | Statistics in Medicine | 2024 | Dirichlet process prior mixture | Bayesian | Code provided in Supplementary |
| A simple and robust method for multivariate meta-analysis of diagnostic test accuracy. | Chen Y | Statistics in Medicine | 2017 | Bivariate and trivariate Sarmanov | Frequentist | Implemented in the R package *xmeta*^5^ |
| The Matrix Stick-Breaking Process: Flexible Bayes Meta-Analysis | Dunson D | Journal of the American Statistical Association | 2008 | Dirichlet process mixture prior | Bayesian | Code not provided |
| Latent class bivariate model for the meta-analysis of diagnostic test accuracy studies | Eusebi P | BMC Medical Research Methodology | 2014 | Mixture of (bivariate) normal distributions | Frequentist | Implemented in the new software^6^ |
| Semiparametric Bayesian Density Estimation With Disparate Data Sources: A Meta-Analysis of Global Childhood Undernutrition | Finucane MM | Journal of the American Statistical Association | 2015 | Mixture of normal distributions | Bayesian | Code not provided |
| A bivariate approach to meta-analysis | Van Houwelingen HC | Statistics in Medicine | 1993 | Mixture algorithm | Frequentist | Code not provided |
| Bayesian semiparametric mixed effects models for meta-analysis of the literature data : An application to cadmium toxicity studies | Jo S | Statistics in Medicine | 2021 | Dirichlet process prior mixture | Bayesian | Code not provided |
| A Bayesian non-parametric Meta-analysis model | Karabatsos G | Research Synthesis Methods | 2013 | Mixture of normal distributions including co-variates | Bayesian | Implemented in the new software^7^ |
| Flexible parametric models for random effects | Lee K | Statistics in Medicine | 2008 | Skew-normal, t- and skewed t-distribution | Bayesian | Code not provided |
| Bayesian meta-analysis of longitudinal data models using Multivariate Mixture priors | Lopes H | Biometrics | 2003 | Mixture of multivariate normal distributions | Bayesian | Code not provided |
| Meta-analysis using Dirichlet process | Muthukumarana S | Statistical Methods in Medical Research | 2012 | Dirichlet process  prior mixture | Bayesian | Code provided in Supplementary |
| Skewed normal RE models for meta-analysis of DTA* studies | Negeri Z | Biometrical Journal | 2019 | Bivariate Skew-normal distribution | Frequentist | Code provided in Supporting information |
| Meta-analysis using flexible Random effects distribution models | Noma H | Journal of epidemiology | 2021 | Skewed extensions of normal and t-distribution | Bayesian | Implemented in the R package *flexmeta*^8^ |
| Flexible random effects models using Bayesian semi-parametric models using Bayesian semi-parametric models: Applications to institutional comparisons | Ohlssen D | Statistics in Medicine | 2007 | Dirichlet Process prior mixture | Bayesian | Code provided in Supplementary |
| Nonparametric estimation of the random effects distribution for the risk or rate ratio in rare events meta-analysis with the arm-based and contrast-based approaches | Sangnawakij P | Statistics in Medicine | 2024 | Mixture algorithm | Frequentist | Code not provided |
| Mixture models in diagnostic meta-analysis**-**Clustering summary receiver operating characteristic curves accounted for heterogeneity and correlation | Schlattmann P | Journal of clinical Epidemiology | 2014 | Mixture of (bivariate) normal distributions | Frequentist | Code provided in Supplementary |
| Disentangling Effect Size Heterogeneity in Meta-Analysis: A Latent Mixture Approach | Zhang N | American Psychological Association | 2020 | Mixture of normal distributions for moderator analysis | Frequentist | Code not provided |
| **Abbreviation: Diagnostic test accuracy studies (DTA)** | | | | | | |

**Supplementary Table 2**. Bayesian models and scenarios included (✓) or excluded (🗶) from the simulation study due to lack of convergence in more than 5% of the datasets.

| **Scenario** | **Models** | | | | | | | | | | |
| --- | --- | --- | --- | --- | --- | --- | --- | --- | --- | --- | --- |
|  | **Binomial-Normal (HN)** | **Binomial-Normal (Unif)** | **Binomial-t(HN)** | **Binomial-t(Unif)** | **Binomial-SN(HN)** | **Binomial-SN(Unif)** | **Binomial-DP-26 (HN/Unif)** | **Binomial-DP-51 (HN/Unif)** | **Binomial-DP-26 (Unif/Unif)** | **Binomial-DP-51 (Unif/Unif)** | **Binomial-DP-**$\boldsymbol{n}$ **(Unif/Gamma)** |
| **Scenario 1** |  |  |  |  |  |  |  |  |  |  |  |
| **Scenario 2** |  |  |  |  |  |  |  |  |  |  |  |
| **Scenario 3** |  |  |  |  |  |  |  |  |  |  |  |
| **Scenario 4** |  |  |  |  |  |  |  |  |  |  |  |
| **Scenario 5** |  |  |  |  |  |  |  |  |  |  |  |
| **Scenario 6** |  |  |  |  |  |  |  |  |  |  |  |
| **Scenario 7** |  |  |  |  |  |  |  |  |  |  |  |
| **Scenario 8** |  |  |  |  |  |  |  |  |  |  |  |
| **Scenario 9** |  |  |  |  |  |  |  |  |  |  |  |
| **Scenario 10** |  |  |  |  |  |  |  |  |  |  |  |
| **Scenario 11** |  |  |  |  |  |  |  |  |  |  |  |
| **Scenario 12** |  |  |  |  |  |  |  |  |  |  |  |
| **Scenario 13** |  |  |  |  |  |  |  |  |  |  |  |
| **Scenario 14** |  |  |  |  |  |  |  |  |  |  |  |
| **Scenario 15** |  |  |  |  |  |  |  |  |  |  |  |
| **Scenario 16** |  |  |  |  |  |  |  |  |  |  |  |
| **Scenario 17** |  |  |  |  |  |  |  |  |  |  |  |
| **Scenario 18** |  |  |  |  |  |  |  |  |  |  |  |
| **Scenario 19** |  |  |  |  |  |  |  |  |  |  |  |
| **Scenario 20** |  |  |  |  |  |  |  |  |  |  |  |
| **Scenario 21** |  |  |  |  |  |  |  |  |  |  |  |
| **Scenario 22** |  |  |  |  |  |  |  |  |  |  |  |

**Supplementary Table 3.** Summary of performance metrics for the common mean mixture model, reporting results separately for outlying and non-outlying studies. The metrics include mean absolute bias, percent relative bias, mean squared error (MSE), and normalized MSE, for both standard deviation components of the model.

|  | **Non-outlying studies (component 1)** | | | | **Outlying studies (component 2)** | | | |
| --- | --- | --- | --- | --- | --- | --- | --- | --- |
| **Scenarios** | **Mean absolute bias of** $\boldsymbol{\tau}_{\boldsymbol{comp}\boldsymbol{1}}$ | **Percent bias of** $\boldsymbol{\tau}_{\boldsymbol{comp}\boldsymbol{1}}$ | **MSE of** $\boldsymbol{\tau}_{\boldsymbol{comp}\boldsymbol{1}}$ | **Normalized MSE of** $\boldsymbol{\tau}_{\boldsymbol{comp}\boldsymbol{1}}$ | **Mean absolute bias of** $\boldsymbol{\tau}_{\boldsymbol{comp}\boldsymbol{2}}$ | **Percent bias of** $\boldsymbol{\tau}_{\boldsymbol{comp}\boldsymbol{2}}$ | **MSE of** $\boldsymbol{\tau}_{\boldsymbol{comp}\boldsymbol{2}}$ | **Normalized MSE of** $\boldsymbol{\tau}_{\boldsymbol{comp}\boldsymbol{2}}$ |
| **Scenario1** | 0.11 | -20.59% | 0.02 | 0.19 | 0.14 | 7.61% | 0.04 | 0.35 |
| **Scenario2** | 0.58 | -30.31% | 0.59 | 0.23 | 0.37 | 4.08% | 0.25 | 0.10 |
| **Scenario3** | 0.12 | -25.3% | 0.03 | 0.21 | 0.14 | 9.45% | 0.04 | 0.34 |
| **Scenario4** | 0.57 | -29.75% | 0.57 | 0.22 | 0.37 | 3.77% | 0.26 | 0.10 |
| **Scenario5** | 0.09 | -15.91% | 0.02 | 0.13 | 0.12 | 0.91% | 0.03 | 0.27 |
| **Scenario6** | 0.47 | -25.03% | 0.44 | 0.17 | 0.28 | 3.64% | 0.17 | 0.06 |
| **Scenario7** | 0.09 | -15.4% | 0.02 | 0.13 | 0.12 | 2.38% | 0.04 | 0.30 |
| **Scenario8** | 0.48 | -25.49% | 0.46 | 0.17 | 0.27 | 4.87% | 0.20 | 0.06 |
| **Scenario9** | 0.13 | -26.51% | 0.03 | 0.23 | 0.17 | 19.73% | 0.07 | 0.55 |
| **Scenario10** | 0.60 | -31.85% | 0.59 | 0.23 | 0.54 | 15.25% | 0.73 | 0.28 |
| **Scenario11** | 0.13 | -26.09% | 0.03 | 0.23 | 0.16 | 17.20% | 0.05 | 0.41 |
| **Scenario12** | 0.56 | -29.14% | 0.53 | 0.20 | 0.48 | 9.04% | 0.55 | 0.21 |
| **Scenario13** | 0.1 | -19.97% | 0.02 | 0.16 | 0.16 | 16.7% | 0.06 | 0.48 |
| **Scenario14** | 0.50 | -26.77% | 0.44 | 0.17 | 0.47 | 16.97% | 0.55 | 0.21 |
| **Scenario15** | 0.09 | -18.59% | 0.02 | 0.14 | 0.16 | 15.84% | 0.06 | 0.49 |
| **Scenario16** | 0.43 | -23.08% | 0.35 | 0.13 | 0.41 | 11% | 0.44 | 0.17 |
| **Scenario17** | 0.25 | -40.64% | 0.10 | 0.41 | 0.23 | 30.93% | 0.10 | 0.40 |
| **Scenario18** | 0.16 | -18.94% | 0.05 | 0.15 | 0.14 | 4.42% | 0.04 | 0.12 |
| **Scenario19** | 0.63 | -38.58 | 0.62 | 0.30 | 0.44 | 12.29% | 0.41 | 0.20 |
| **Scenario20** | 0.21 | -32.26% | 0.08 | 0.33 | 0.20 | 27.45% | 0.08 | 0.33 |
| **Scenario21** | 0.12 | -13.73% | 0.03 | 0.10 | 0.11 | 3.93% | 0.03 | 0.09 |
| **Scenario22** | 0.60 | -38.38% | 0.58 | 0.28 | 0.38 | 15.53% | 0.30 | 0.15 |
| $\boldsymbol{\tau}_{\boldsymbol{comp}\boldsymbol{1}}$**= the standard deviation of non-outlying studies,** $\boldsymbol{\tau}_{\boldsymbol{comp}\boldsymbol{2}}$**= the standard deviation of outlying studies** | | | | | | | | |

1. **Skewness coefficient and concentration parameter**

For the binomial-SN models we used a vague asymmetric prior$N(0,25)$, for the shape parameter ($a$), as suggested by Noma et al.^8^ that does not impose any direction for the mean of the random-effects distribution. Such a prior reflects a range for $a$ around [-27, 27] being in accordance with the skewness coefficient’s $(\gamma$) restricted range around [-0.99, 0.99]^9^. Supplementary Table 4 shows the skewness coefficient as this was estimated from the binomial-SN(HN) and the binomial-SN(Unif) models when data were generated from a skew-normal distribution. The mean absolute bias of $\gamma$ in case of binomial-SN(HN) model ranged from 0.36 to 0.53 with the smallest values to be attributed in scenarios with 26 number of studies and in case of binomial-SN(Unif) model the bias was ranged from 0.22 to 0.52 and was generally larger for scenarios with moderate random-effects variance. Supplementary Table 5 presents the mean of the concentration parameter from the various binomial-DP models that can be used as an indicator of the shape of the random effects’ distribution. When we used a uniform prior for $\alpha$, the estimated mean was always larger than the mean of the respective prior distribution for true distributions closer to the normal or skew normal indicating underlying effects distribution closer to the normal base distribution. On the other hand, the concentration parameter was always smaller than the mean of the prior distribution when the true distribution was closer to a bimodal distribution indicating non-normal underlying effects distribution. Similarly, when a prior $\Gamma(1,1)$ was used, the estimated mean of the concentration parameter constantly exceeded the highly compressed range employed by the respective prior for true distributions closer to the normal or skew normal, while it did not converge when the true distribution was bimodal. However, Top of Form the estimation of this parameter was generally imprecise probably due to the relatively small number of studies we used in the meta-analyses.

**Supplementary Table 4.** Mean absolute bias of the estimated skewness coefficient ($\gamma$) from the skew-normal model in scenarios where data were generated from a skew-normal distribution

| **Scenario** | **Mean absolute bias** | |
| --- | --- | --- |
|  | **Binomial-SN(HN)** | **Binomial-SN(Unif)** |
| **Scenario 9** | 0.53 | 0.52 |
| **Scenario 10** | 0.51 | 0.36 |
| **Scenario 11** | 0.53 | 0.52 |
| **Scenario 12** | 0.52 | 0.37 |
| **Scenario 13** | 0.38 | 0.38 |
| **Scenario 14** | 0.39 | 0.23 |
| **Scenario 15** | 0.36 | 0.35 |
| **Scenario 16** | 0.38 | 0.22 |

**Supplementary Table 5**. Estimated means with credible intervals of the concentration parameter ($\alpha$) from the five DP models for the different simulation scenarios.

| **Scenario** | **True distribution** | **Number of studies** | **Model** | | | | |
| --- | --- | --- | --- | --- | --- | --- | --- |
|  |  |  | Binomial-DP-26(HN/Unif) | Binomial-DP-51(HN/Unif) | Binomial-DP-26(Unif/Unif) | Binomial-DP-51(Unif/Unif) | Binomial-DP-n(Unif/Gamma) |
| **Scenario 1** | $N\left( 0,0.12 \right)$ | 14 | 2.89 [0.57, 4.90] | Non-convergence | Non-convergence | Non-convergence | Non-convergence |
| **Scenario 2** | $N\left( 0,2.63 \right)$ | 14 | 3.89 [1.58, 4.95] | 7.31[2.64,9.87] | 3.81[1.49,4.95] | 7.10[2.43,9.86] | 2.47[0.68,5.93] |
| **Scenario 3** | $N\left( 0.5,0.12 \right)$ | 14 | Non-convergence | Non-convergence | Non-convergence | Non-convergence | Non-convergence |
| **Scenario 4** | $N\left( 0.5,2.63 \right)$ | 14 | 3.89 [1.58, 4.95] | 7.31[2.65,9.87] | 3.81[1.49,4.95] | 7.11[2.45,9.86] | 2.46[0.67,5.92] |
| **Scenario 5** | $N\left( 0,0.12 \right)$ | 26 | 3.08 [0.66, 4.90] | 5.80[1.09, 9.78] | Non-convergence | Non-convergence | Non-convergence |
| **Scenario 6** | $N\left( 0,2.63 \right)$ | 26 | 4.16 [2.10, 4.96] | 7.95[3.57,9.91] | 4.12[2.00,4.96] | 7.83[3.38,9.90] | 3.41[1.21,7.45] |
| **Scenario 7** | $N\left( 0.5,0.12 \right)$ | 26 | 3.09 [0.68, 4.90] | 5.86[1.14, 9,79] | Non-convergence | Non-convergence | Non-convergence |
| **Scenario 8** | $N\left( 0.5,2.63 \right)$ | 26 | 4.18 [2.13, 4.97] | 7.99[3.65,9.91] | 4.13[2.03,4.96] | 7.85[3.43,9.90] | 3.48[1.25,7.53] |
| **Scenario 9** | $\mathrm{SN}\left( 0,0.12,0.785 \right)$ | 14 | Non-convergence | Non-convergence | Non-convergence | Non-convergence | Non-convergence |
| **Scenario 10** | $\mathrm{SN}\left( 0,2.63,0.785 \right)$ | 14 | 3.84 [1.51, 4.95] | 7.19[2.49,9.87] | 3.77[1.43,4.94] | 6.99[2.32,9.85] | 2.39[0.63,5.85] |
| **Scenario 11** | $\mathrm{SN}\left( 0.5,0.12,0.785 \right)$ | 14 | Non-convergence | Non-convergence | Non-convergence | Non-convergence | Non-convergence |
| **Scenario 12** | $\mathrm{SN}\left( 0.5,2.63,0.785 \right)$ | 14 | 3.85 [1.53, 4.95] | 7.18[2.50,9.87] | 3.78[1.44,4.94] | 6.99[2.35,9.85] | 2.40[0.63,5.87] |
| **Scenario 13** | $\mathrm{SN}\left( 0,0.12,0.785 \right)$ | 26 | 3.01 [0.64, 4.90] | Non-convergence | Non-convergence | Non-convergence | Non-convergence |
| **Scenario 14** | $\mathrm{SN}\left( 0,2.63,0.785 \right)$ | 26 | 4.12 [2.02, 4.96] | 7.80[3.39,9.90] | 4.07[1.93,4.96] | 7.67[3.20,9.89] | 3.28[1.15,7.26] |
| **Scenario 15** | $\mathrm{SN}\left( 0.5,0.12,0.785 \right)$ | 26 | 3.00 [0.64, 4.90] | 5.61[1.04,9.76] | Non-convergence | Non-convergence | Non-convergence |
| **Scenario 16** | $\mathrm{SN}\left( 0.5,2.63,0.785 \right)$ | 26 | 4.12 [2.01, 4.96] | 7.82[3.39,9.90] | 4.07[1.93,4.96] | 7.66 [3.19,9.89] | 3.27[1.14,7.24] |
| **Scenario 17** | $0.3N\left( 0,0.12 \right)$+$0.7N\left( 1,0.005 \right)$ | 14 | 2.54 [0.52, 4.84] | 4.34[0.70,9.58] | Non-convergence | Non-convergence | Non-convergence |
| **Scenario 18** | $0.3N\left( 0,0.12 \right)$+$0.7N\left( 1,0.12 \right)$ | 14 | 3.25 [0.79, 4.91] | 5.98[1.28,9.79] | Non-convergence | Non-convergence | Non-convergence |
| **Scenario 19** | $0.3N\left( 0,0.12 \right)$+$0.7N\left( 1,2.63 \right)$ | 14 | 3.76 [1.42, 4.94] | 6.97[2.32,9.85] | 3.67[1.32,4.94] | 6.71[2.11,9.82] | 2.24[0.54,5.64] |
| **Scenario 20** | $0.3N\left( 0,0.12 \right)$+$0.7N\left( 1,0.005 \right)$ | 26 | 2.53 [0.55, 4.82] | 3.93[0.71,9.35] | Non-convergence | Non-convergence | Non-convergence |
| **Scenario 21** | $0.3N\left( 0,0.12 \right)$+$0.7N\left( 1,0.12 \right)$ | 26 | 3.48 [1.01, 4.93] | 6.42[1.66,9.82] | Non-convergence | Non-convergence | Non-convergence |
| **Scenario 22** | $0.3N\left( 0,0.12 \right)$+$0.7N\left( 1,2.63 \right)$ | 26 | 4.05 [1.90, 4.96] | 7.59[3.12,9.89] | 3.99[1.80,4.96] | 7.45[2.95,9.88] | 3.05[1.02,6.92] |

**Supplementary Table 6**. Mean and variance of the random-effects distribution estimates along with their 95% Confidence/Credible Intervals for each selected simulated dataset using all models evaluated in the simulation study. The 95% prediction intervals are also presented.

| **Scenario 6** | | | |
| --- | --- | --- | --- |
| **Models** | **Mean of the random-effects distribution 95% Confidence/Credible Intervals** | **Random-effects variance 95% Confidence/Credible Intervals** | **Prediction 95%Prediction Intervals** |
| **Binomial-Normal(HN)** | 0.14 [-0.36, 0.62] | 1.54 [0.90, 2.73] | 0.15 [-2.48, 2.64] |
| **Binomial-Normal(Unif)** | 0.15 [-0.41, 0.65] | 1.69 [0.97, 3.18] | 0.14 [-2.61, 2.86] |
| **Binomial-t(HN)** | 0.15 [-0.27, 0.57] | 1.71 [1.01, 3.27] | 0.15 [-2.04, 2.34] |
| **Binomial-t(Unif)** | 0.15 [-0.28,0.59] | 1.83 [1.07, 3.58] | 0.16 [-2.14, 2.45] |
| **Binomial-SN(HN)** | 0.15 [-0.27, 0.57] | 1.54 [0.98, 2.52] | 0.16 [-1.98, 2.24] |
| **Binomial-SN(Unif)** | 0.15 [-0.32, 0.61] | 1.83 [1.10, 3.29] | 0.16 [-2.17, 2.38] |
| **Binomial-DP-26(HN/Unif)** | 0.16 [-0.31, 0.63] | 1.46 [0.92, 2.45] | - |
| **Binomial-DP-51(HN/Unif)** | 0.16 [-0.30, 0.65] | 1.48 [0.91, 2.56] | - |
| **Binomial-DP-26(Unif/Unif)** | 0.15 [-0.33, 0.64] | 1.54 [0.95, 2.94] | - |
| **Binomial-DP-51(Unif/Unif)** | 0.16 [-0.35, 0.66] | 1.60 [0.93, 3.01] | - |
| **Binomial-DP-n(Unif/Gamma)** | 0.16 [-0.34, 0.64] | 1.57 [0.93, 2.96] | - |
| **Binomial-Normal(ML)** | 0.16 [-0.31, 0.63] | 1.44 | - |
| **Normal-Normal(REML)** | 0.15 [-0.33, 0.63] | 1.50 [0.91, 2.98] | 0.15 [-2.30, 2.60] |
| **Normal-t** | 0.15 [-0.34, 0.64] | 1.44 | - |
| **Common-mean-mixture** | 0.15 [-0.34, 0.64] | 1.44 | - |
| **Scenario 13** | | | |
| **Models** | **Mean of the random-effects distribution 95% Confidence/Credible Intervals** | **Random-effects variance 95% Confidence/Credible Intervals** | **Prediction 95%Prediction Intervals** |
| **Binomial-Normal(HN)** | -0.13 [-0.35, 0.07] | 0.21 [0.10, 0.45] | -0.13 [-1.08, 0.87] |
| **Binomial-Normal(Unif)** | -0.14 [-0.35, 0.08] | 0.21 [0.10, 0.47] | -0.14 [-1.10, 0.84] |
| **Binomial-t(HN)** | -0.17 [-0.34, 0.01] | 0.22 [0.10, 0.48] | -0.17 [-0.95, 0.62] |
| **Binomial-t(Unif)** | -0.17 [-0.34, 0.01] | 0.22 [0.10, 0.49] | -0.17 [-0.95, 0.63] |
| **Binomial-SN(HN)** | -0.11 [-0.27, 0.06] | 0.18 [0.09, 0.35] | -0.18 [-0.72, 0.71] |
| **Binomial-SN(Unif)** | -0.11 [-0.27, 0.07] | 0.18 [0.09, 0.38] | -0.17 [-0.73, 0.73] |
| **Binomial-DP-26(HN/Unif)** | -0.12 [-0.30, 0.11] | 0.19 [0.08, 0.50] | - |
| **Binomial-DP-51(HN/Unif)** | -0.12 [-0.33, 0.10] | 0.20 [0.08, 0.52] | - |
| **Binomial-DP-26(Unif/Unif)** | -0.12 [-0.34, 0.14] | 0.21 [0.08, 1.33] | - |
| **Binomial-DP-51(Unif/Unif)** | -0.12 [-0.33, 0.13] | 0.21 [0.08, 0.76] | - |
| **Binomial-DP-n(Unif/Gamma)** | -0.13 [-0.35, 0.11] | 0.19 [0.06, 1.37] | - |
| **Binomial-Normal(ML)** | -0.13 [-0.32, 0.06] | 0.18 | - |
| **Normal-Normal(REML)** | -0.13 [-0.32, 0.07] | 0.18 [0.09, 0.47] | -0.13 [-0.99, 0.74] |
| **Normal-t** | -0.20 [-0.38, 0.04] | 0.07 | - |
| **Common-mean-mixture** | -0.27 [-0.41, -0.01] | 0.52 | - |
| **Scenario 20** | | | |
| **Models** | **Mean of the random-effects distribution 95% Confidence/Credible Intervals** | **Random-effects variance 95% Confidence/Credible Intervals** | **Prediction 95%Prediction Intervals** |
| **Binomial-Normal(HN)** | 0.72 [0.48, 0.96] | 0.33 [0.18, 0.65] | 0.72 [-0.49, 1.87] |
| **Binomial-Normal(Unif)** | 0.71 [0.46, 0.96] | 0.33 [0.18, 0.68] | 0.71 [-0.54, 1.93] |
| **Binomial-t(HN)** | 0.75 [0.54, 0.96] | 0.36 [0.20, 0.72] | 0.75 [-0.26, 1.76] |
| **Binomial-t(Unif)** | 0.75 [0.54, 0.96] | 0.36 [0.20, 0.74] | 0.75 [-0.27, 1.78] |
| **Binomial-SN(HN)** | 0.70 [0.50, 0.87] | 0.28 [0.16, 0.51] | 0.78 [-0.34, 1.43] |
| **Binomial-SN(Unif)** | 0.69 [0.48, 0.87] | 0.29 [0.17, 0.55] | 0.78 [-0.38, 1.44] |
| **Binomial-DP-26(HN/Unif)** | 0.70 [0.45, 0.91] | 0.30 [0.17, 0.61] | - |
| **Binomial-DP-51(HN/Unif)** | 0.70 [0.45, 0.91] | 0.30 [0.17, 0.61] | - |
| **Binomial-DP-26(Unif/Unif)** | 0.71 [0.44, 0.93] | 0.32 [0.17, 1.22] | - |
| **Binomial-DP-51(Unif/Unif)** | 0.70 [0.42, 0.93] | 0.32 [0.16, 1.01] | - |
| **Binomial-DP-n(Unif/Gamma)** | 0.72 [0.43, 0.94] | 0.31 [0.16, 2.09] | - |
| **Binomial-Normal(ML)** | 0.71 [0.48, 0.93] | 0.29 | - |
| **Normal-Normal(REML)** | 0.71 [0.48, 0.94] | 0.30 [0.16, 0.60] | 0.71 [-0.39, 1.81] |
| **Normal-t** | 0.71 [0.48, 0.97] | 0.29 | - |
| **Common-mean-mixture** | 0.71 [0.52, 1.15] | 0.29 | - |

**Supplementary Table 7.** Cluster probabilities for each selected simulated dataset using the respective DP model.

| **Probability of cluster’s creation** | | | |
| --- | --- | --- | --- |
| **True distribution** | $\mathbf{N}\boldsymbol{(0, 2.63)}$ | $\mathbf{SN}\boldsymbol{(0, 0.12, 0.785)}$ | $\boldsymbol{0.3}\mathbf{N}\left( \boldsymbol{0,0.12} \right)\boldsymbol{+0.7}\mathbf{N}\boldsymbol{(1,0.005)}$ |
| **Models** | **Binomial-DP-n(Unif/Gamma)** | **Binomial-DP-26(HN/Unif)** | **Binomial-DP-51(HN/Unif)** |
| **Clusters** | **Cluster probabilities** | **Cluster probabilities** | **Cluster probabilities** |
| 1 | **0.12139353** | **0.32834442** | **0.12995617** |
| 2 | **0.11148353** | **0.18654071** | **0.18785258** |
| 3 | **0.1039276** | **0.13384481** | **0.25055638** |
| 4 | **0.09305627** | 0.0904647 | 0.08892688 |
| 5 | **0.08630333** | 0.06191739 | 0.06632126 |
| 6 | **0.07693996** | 0.04830683 | 0.04969604 |
| 7 | **0.0652856** | 0.03435423 | 0.03832933 |
| 8 | **0.05541076** | 0.02658796 | 0.03056589 |
| 9 | 0.04747426 | 0.01999449 | 0.02465271 |
| 10 | 0.03917945 | 0.01563969 | 0.0205349 |
| 11 | 0.03366659 | 0.01154671 | 0.01697345 |
| 12 | 0.02784228 | 0.00879499 | 0.01397231 |
| 13 | 0.02303843 | 0.00696415 | 0.01117701 |
| 14 | 0.01948164 | 0.00533117 | 0.00943516 |
| 15 | 0.01531643 | 0.00449537 | 0.00865246 |
| 16 | 0.01296148 | 0.00343739 | 0.00719769 |
| 17 | 0.0108279 | 0.00291092 | 0.00623596 |
| 18 | 0.00899015 | 0.00208033 | 0.00499309 |
| 19 | 0.00735637 | 0.00168696 | 0.00449656 |
| 20 | 0.00616769 | 0.00136639 | 0.0037815 |
| 21 | 0.00516023 | 0.00104137 | 0.00308059 |
| 22 | 0.00433762 | 0.00083165 | 0.00262279 |
| 23 | 0.00380211 | 0.00068053 | 0.00244143 |
| 24 | 0.00319303 | 0.0005201 | 0.00219439 |
| 25 | 0.00261682 | 0.00041111 | 0.00186492 |
| 26 | 0.01478696 | 0.00190564 | 0.00167222 |
| 27 & more | - | - | 0… |


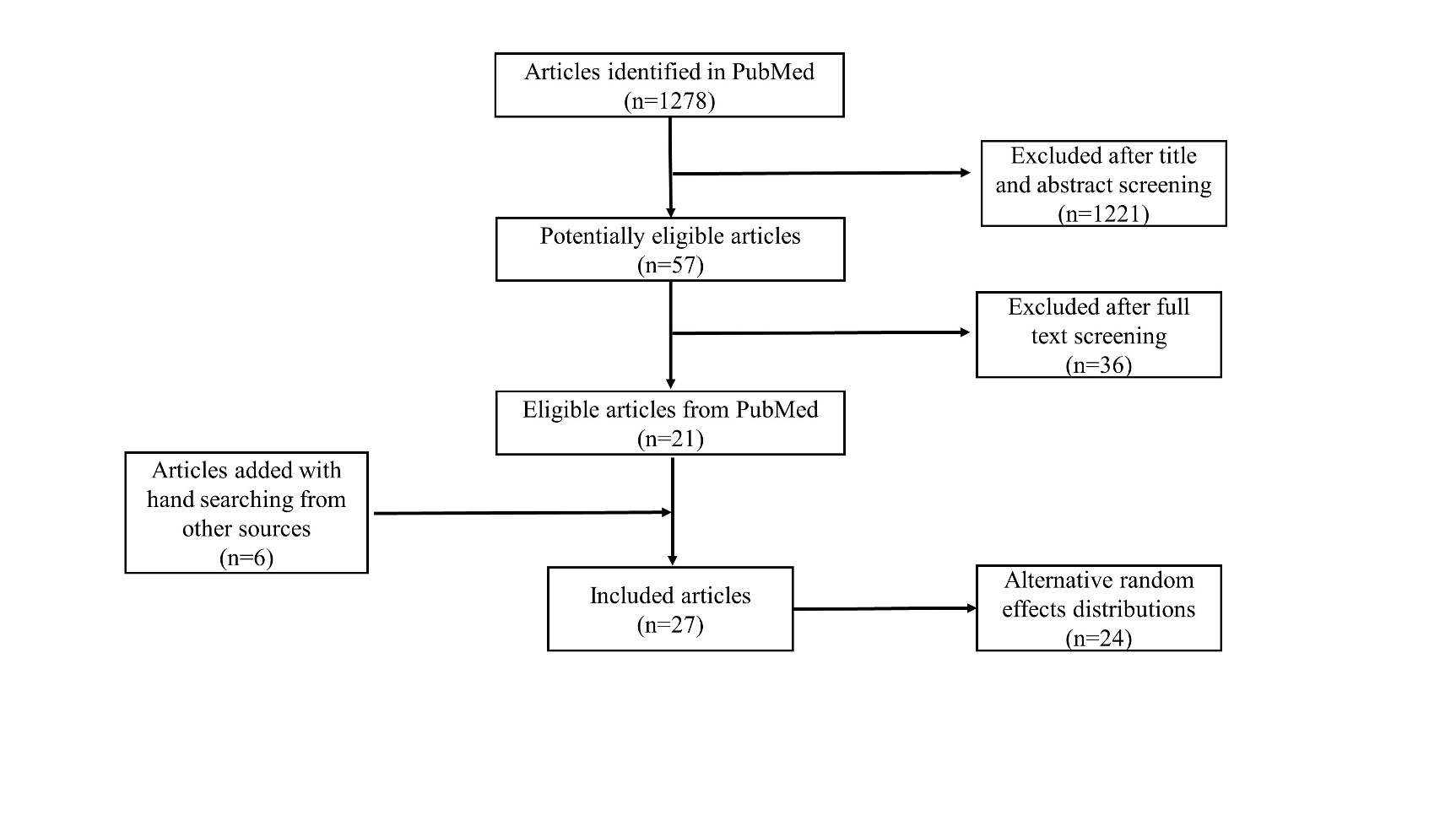


**Supplementary Figure 1**. Flow chart of the article selection process.


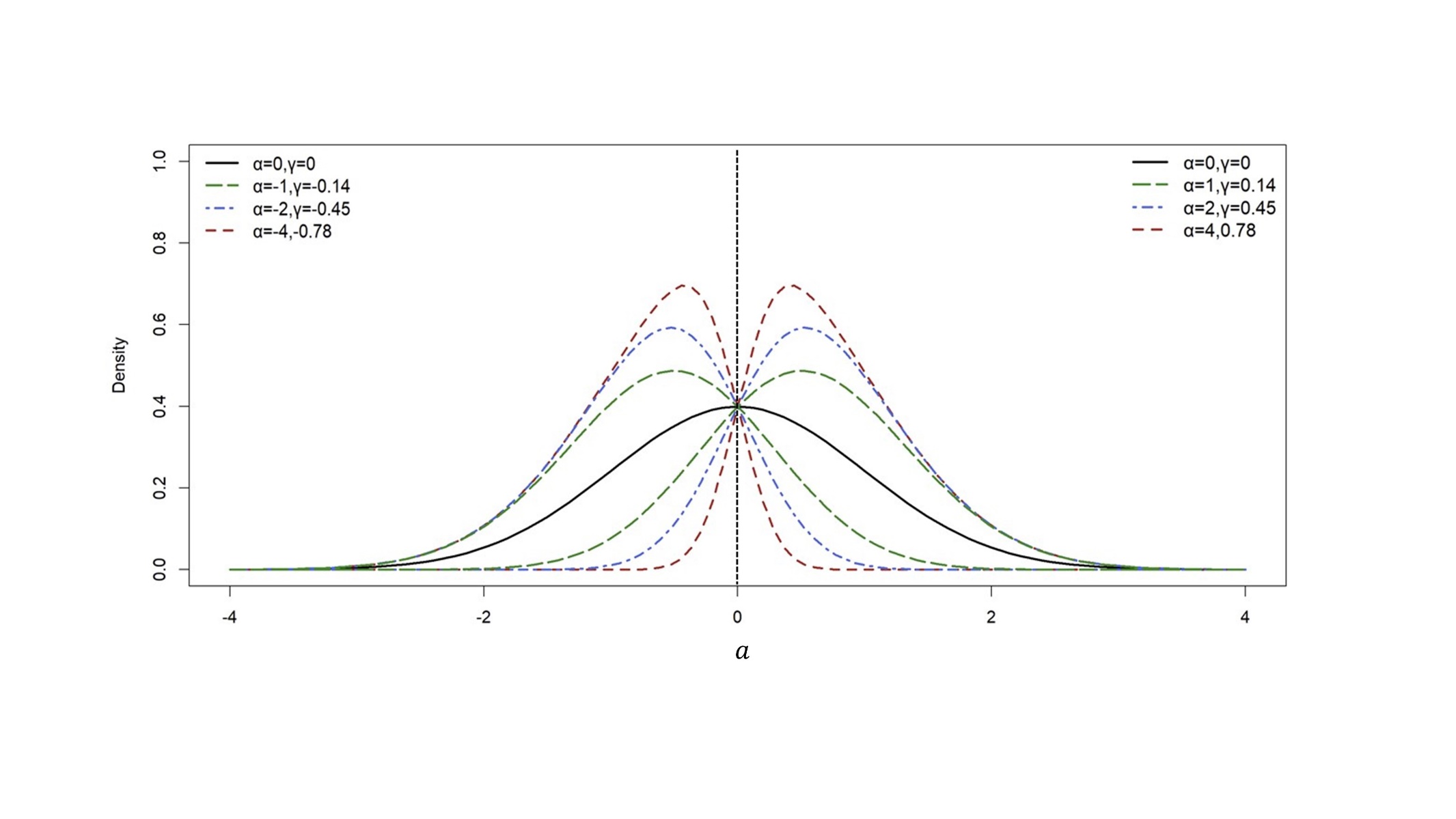
**Supplementary Figure 2.** Density plot of the skew normal distribution with location parameter $\xi=0$, scale parameter $\omega=1$ and various shape parameters ($a$)/skewness coefficients ($\gamma$).


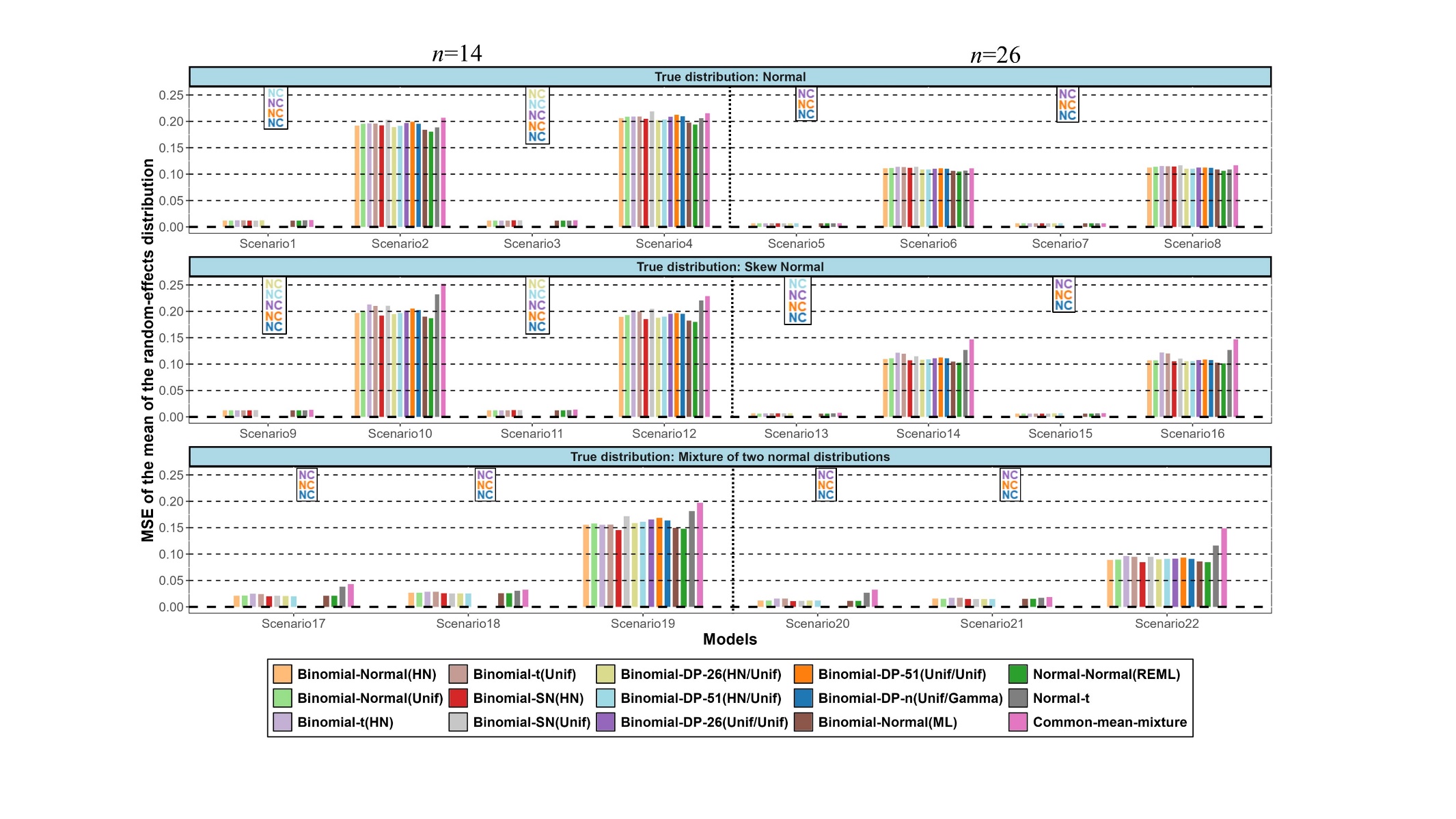
**Supplementary Figure 3.** Simulation results in terms of mean square error (MSE) for the mean of the random-effects distribution. The names of the models are explained in Table 2. (NC=Non-convergence).


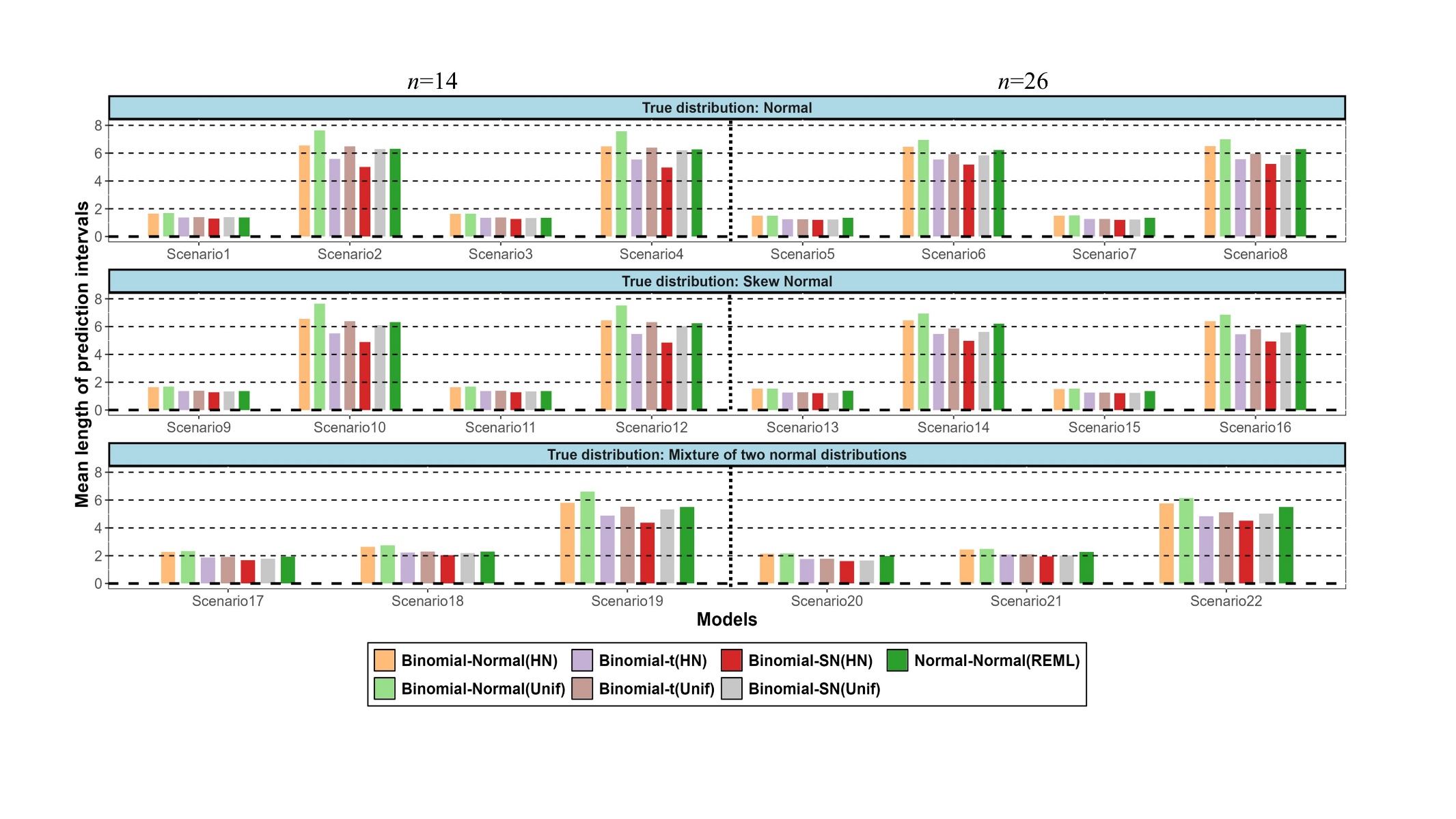
**Supplementary Figure 4.** Simulation results in terms of the mean length of the prediction intervals. The names of the models are explained in Table 2.


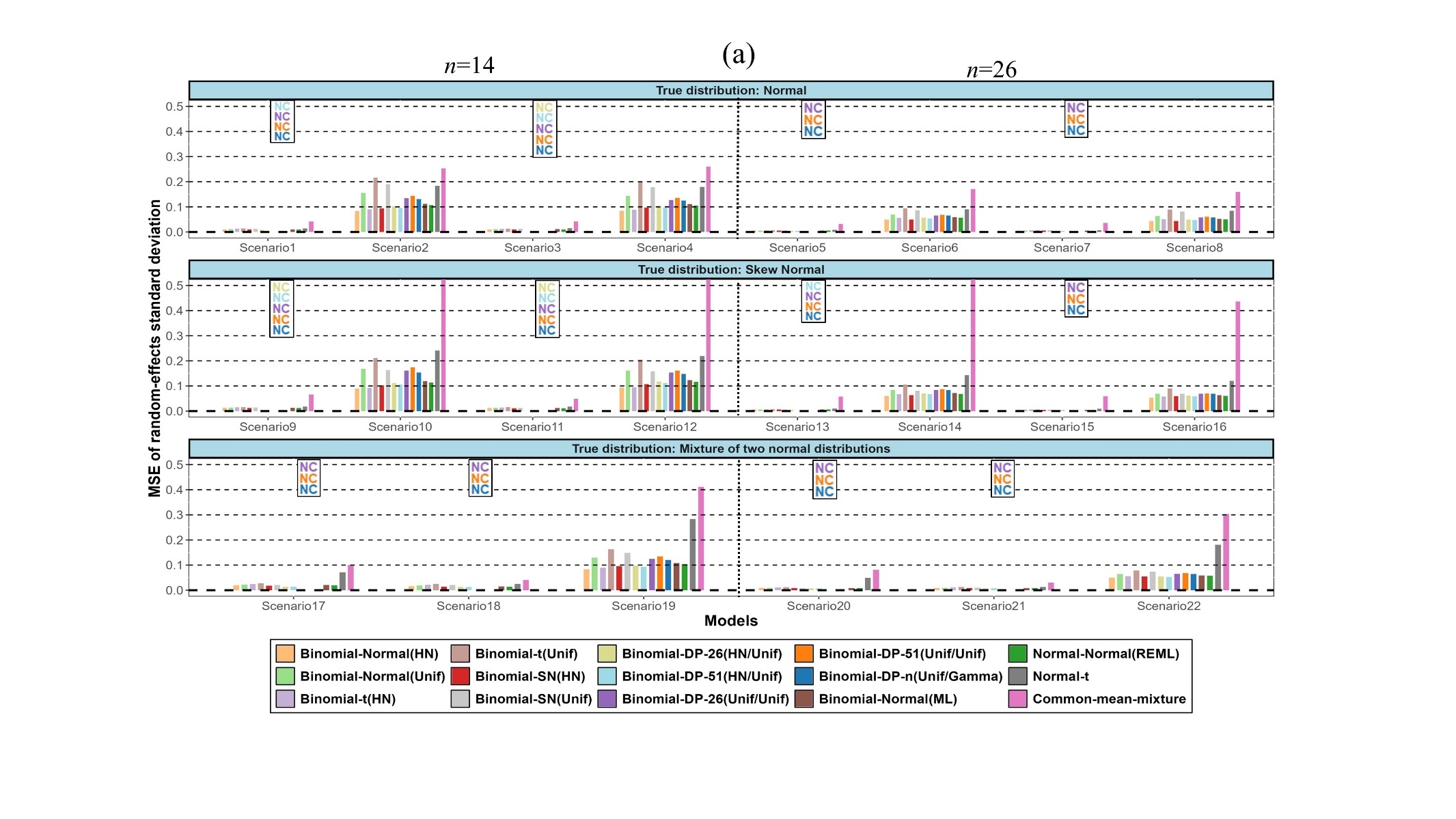


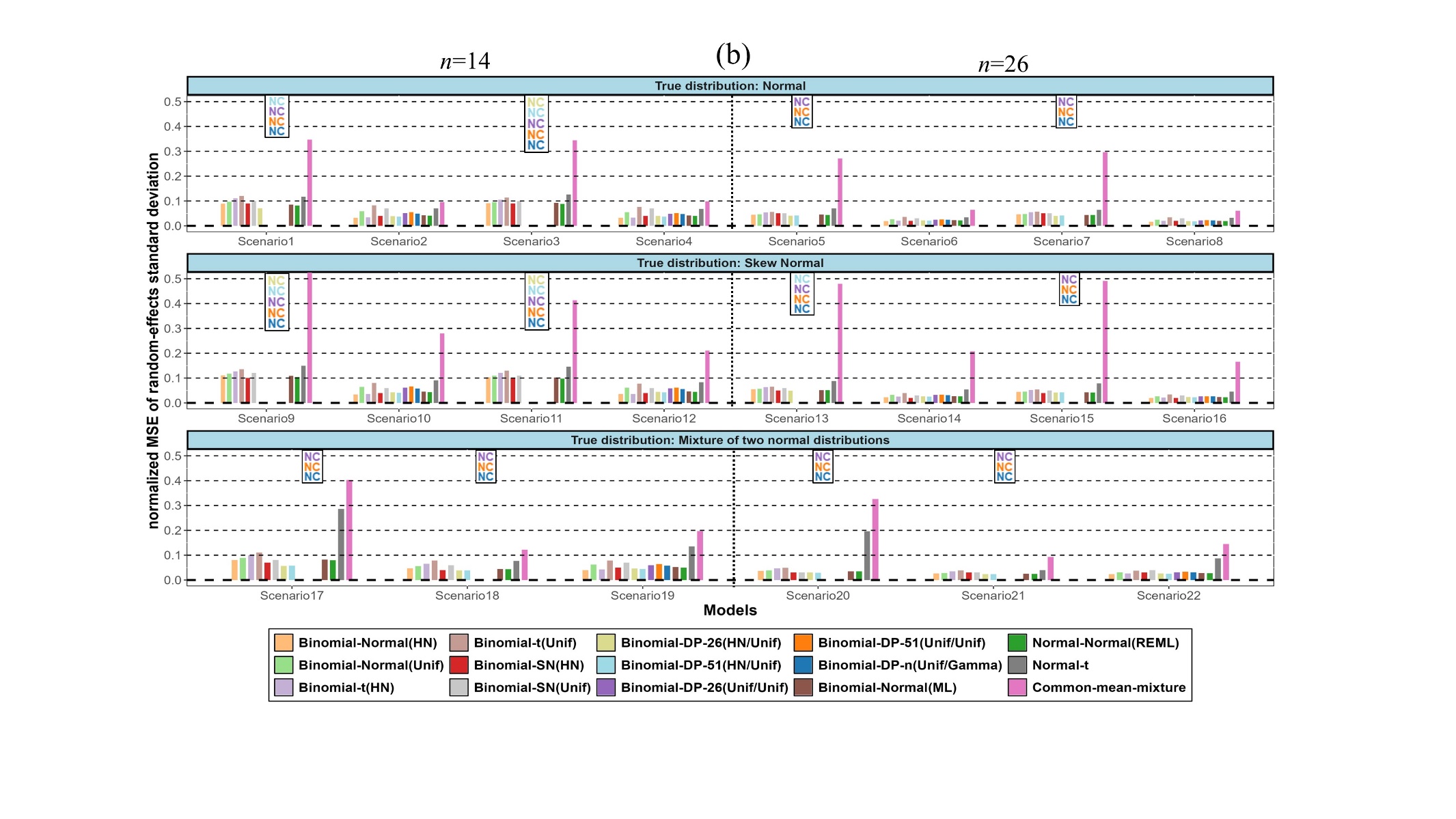
**Supplementary Figure 5**. Simulation results in terms of mean square error (MSE) (a) and normalized mean square error (MSE) (b) for the random-effects standard deviation. The names of the models are explained in Table 2. (NC=Non-convergence)


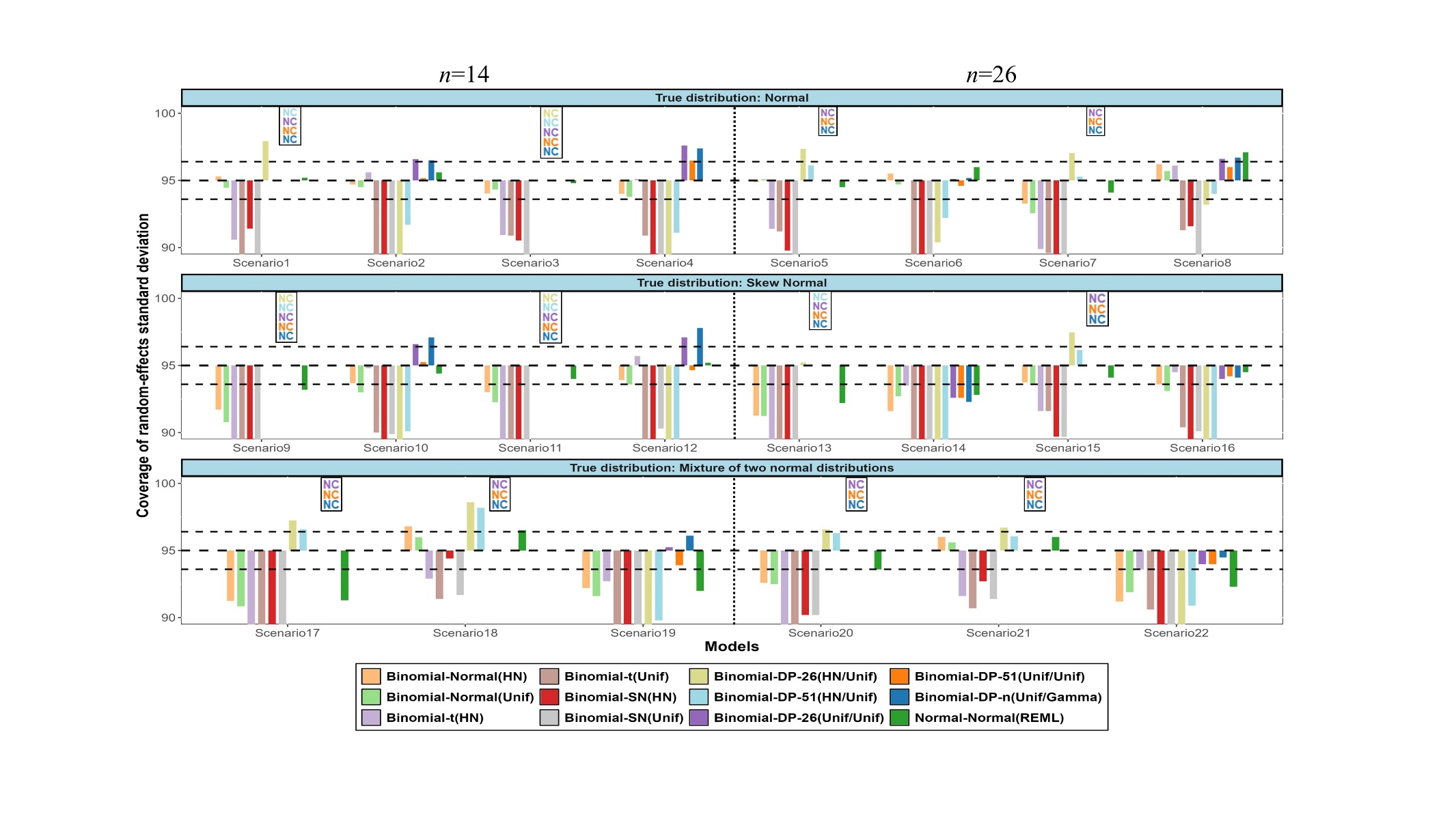
**Supplementary Figure 6.** Simulation results in terms of coverage probability for the random-effects standard deviation. The horizontal lines represent the upper and lower bounds of the 95% confidence interval for the nominal level. The names of the models are explained in Table 2. (NC=Non-convergence).


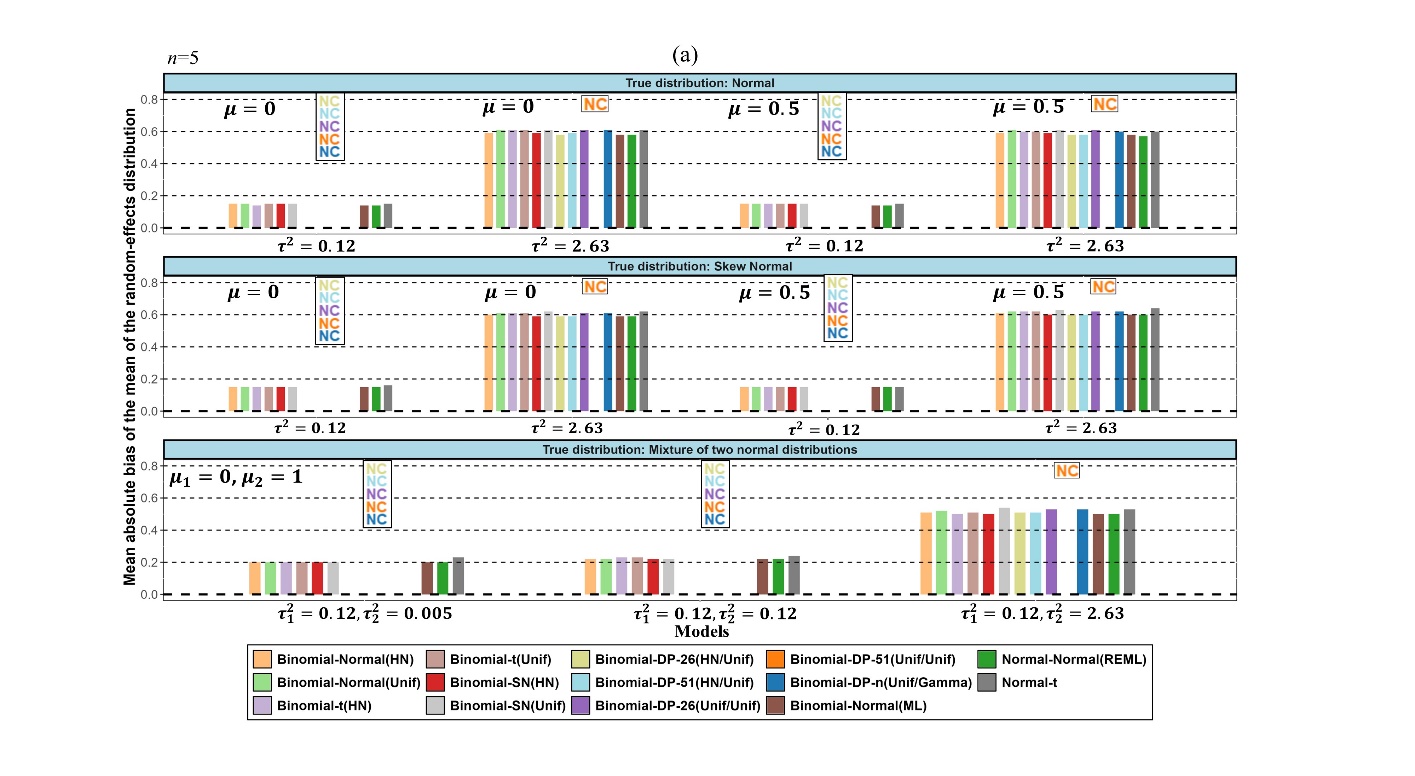


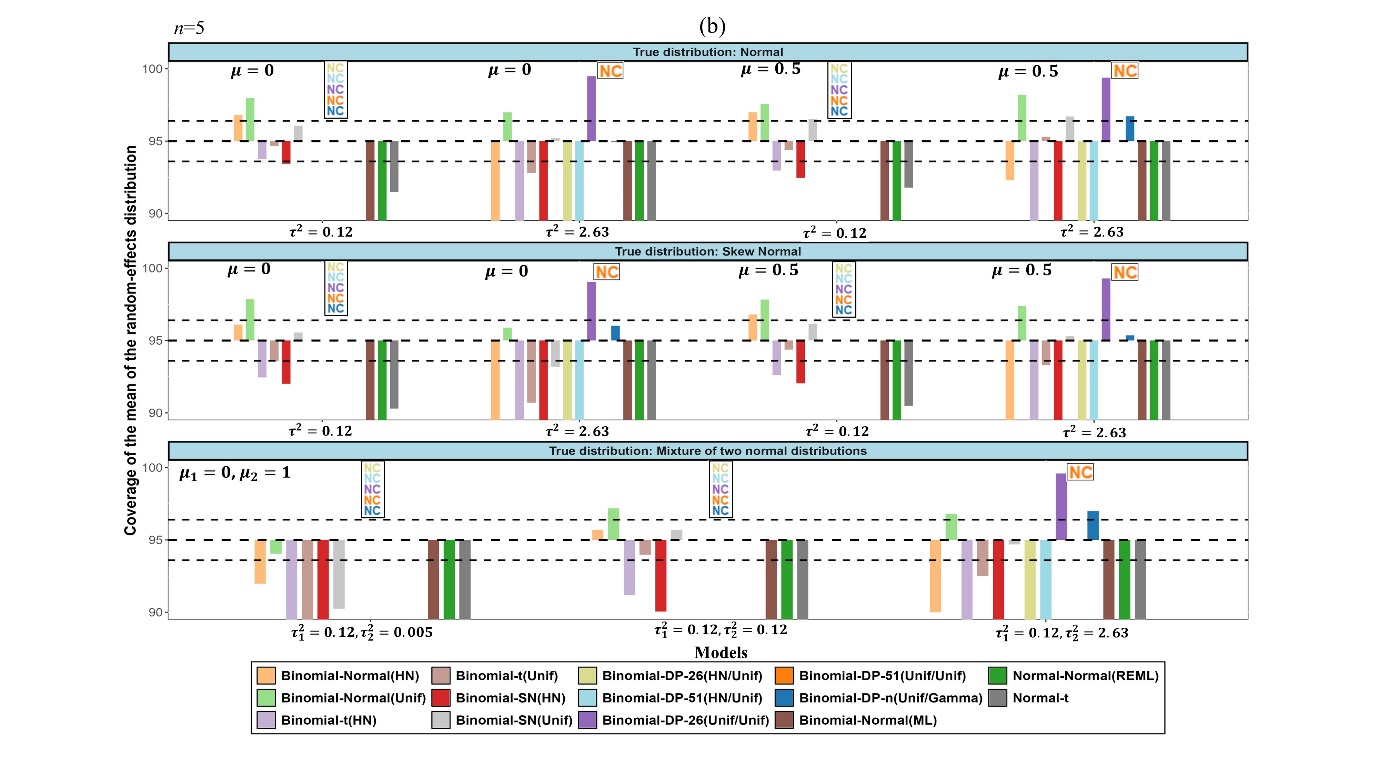


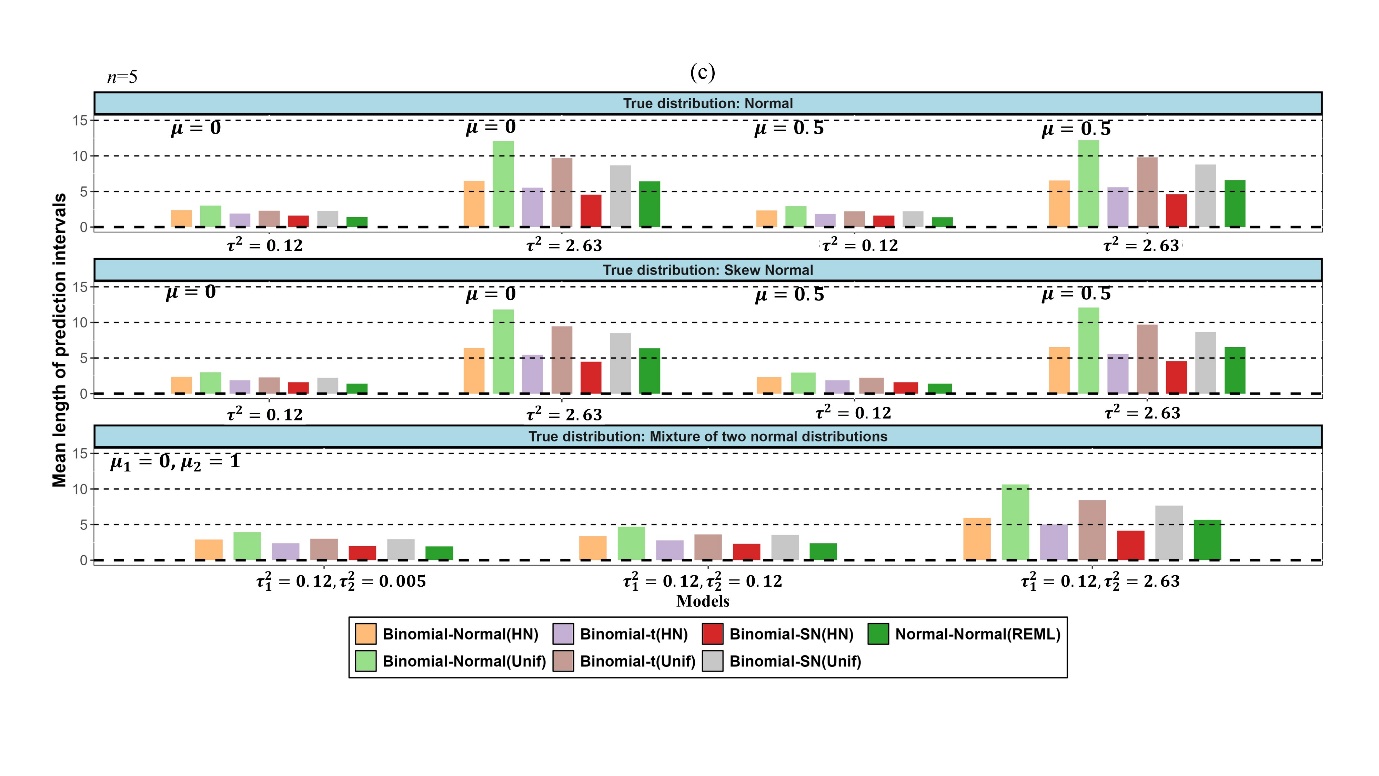


**Supplementary Figure 7**. Simulation results for small meta-analyses (with $n=5$ studies) in terms of mean of absolute bias (a) coverage probability (b) and mean length of the prediction intervals (c) for the mean of the random-effects distribution. Different $\mu,\mu_{1},\mu_{2},\tau^{2},\tau_{1}^{2}$ and $\tau_{2}^{2}$ values represent the true mean(s) and true variance(s) of the different random-effects distributions used for the data generating process. The horizontal lines represent the upper and lower bounds of the 95% confidence interval for the nominal level. The names of the models are explained in Table 2. (NC=Non-convergence)


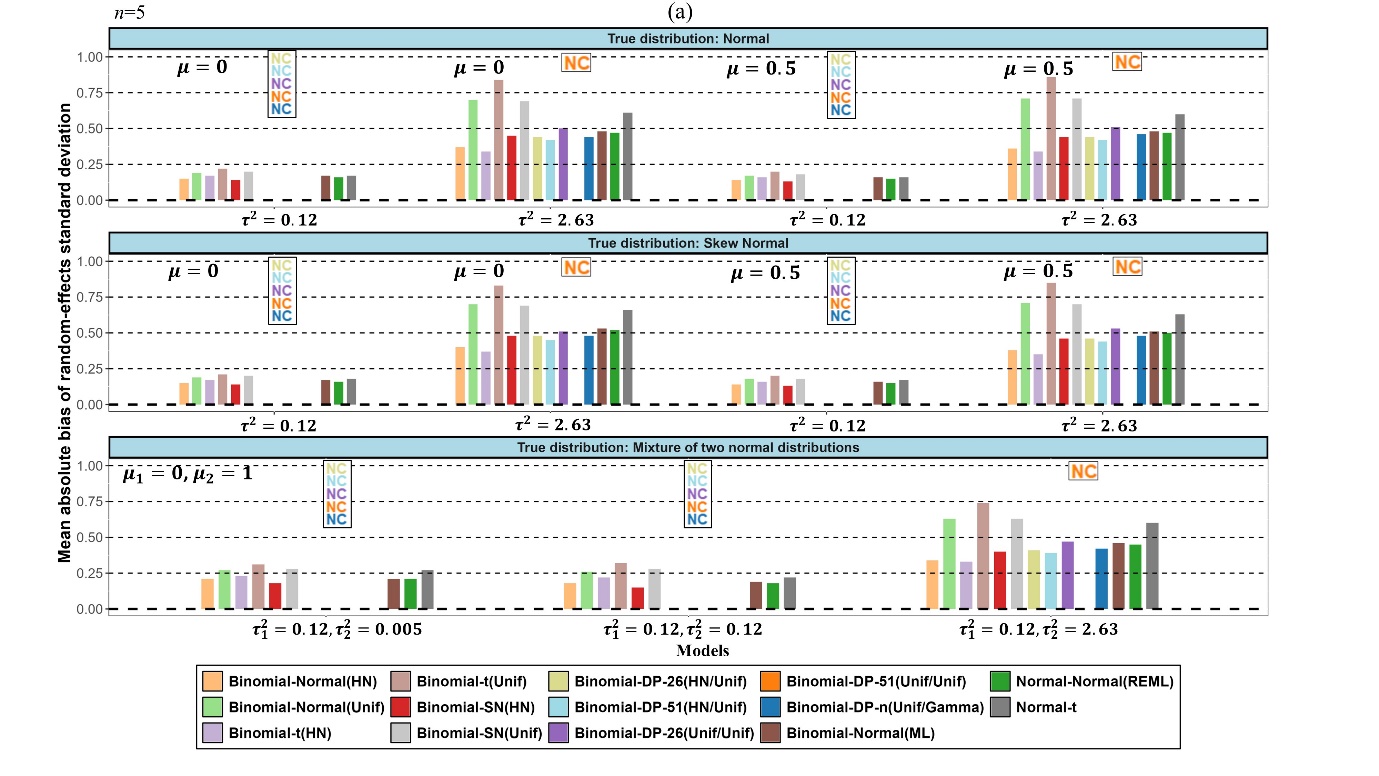


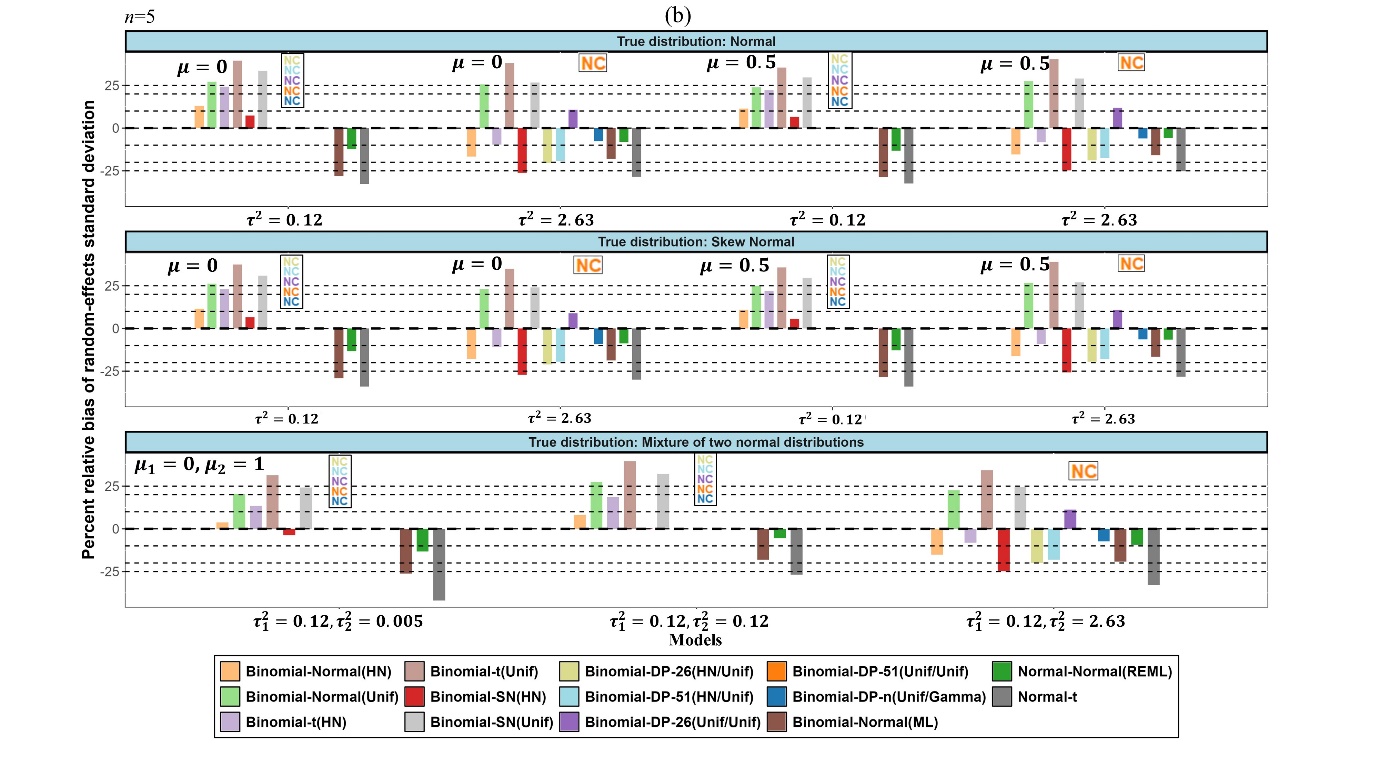


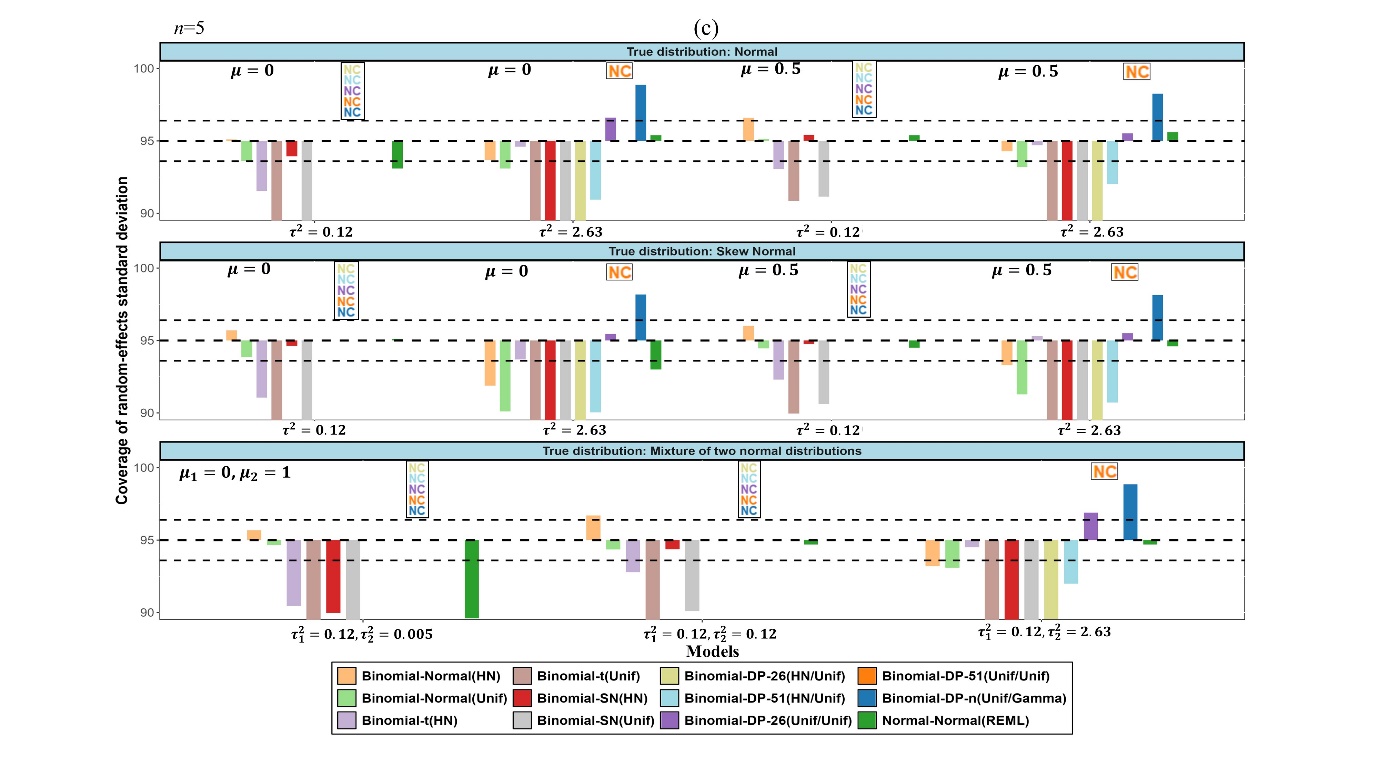
**Supplementary Figure 8**. Simulation results for small meta-analyses (with $n=5$ studies) in terms of mean of absolute bias (a) percent relative bias (b) and coverage probability (c) for the random-effects standard deviation. Different $\mu,\mu_{1},\mu_{2},\tau^{2},\tau_{1}^{2}$ and $\tau_{2}^{2}$ values represent the true mean(s) and true variance(s) of the different random-effects distributions used for the data generating process. The horizontal lines represent the upper and lower bounds of the 95% confidence interval for the nominal level. The names of the models are explained in Table 2. (NC=Non-convergence)


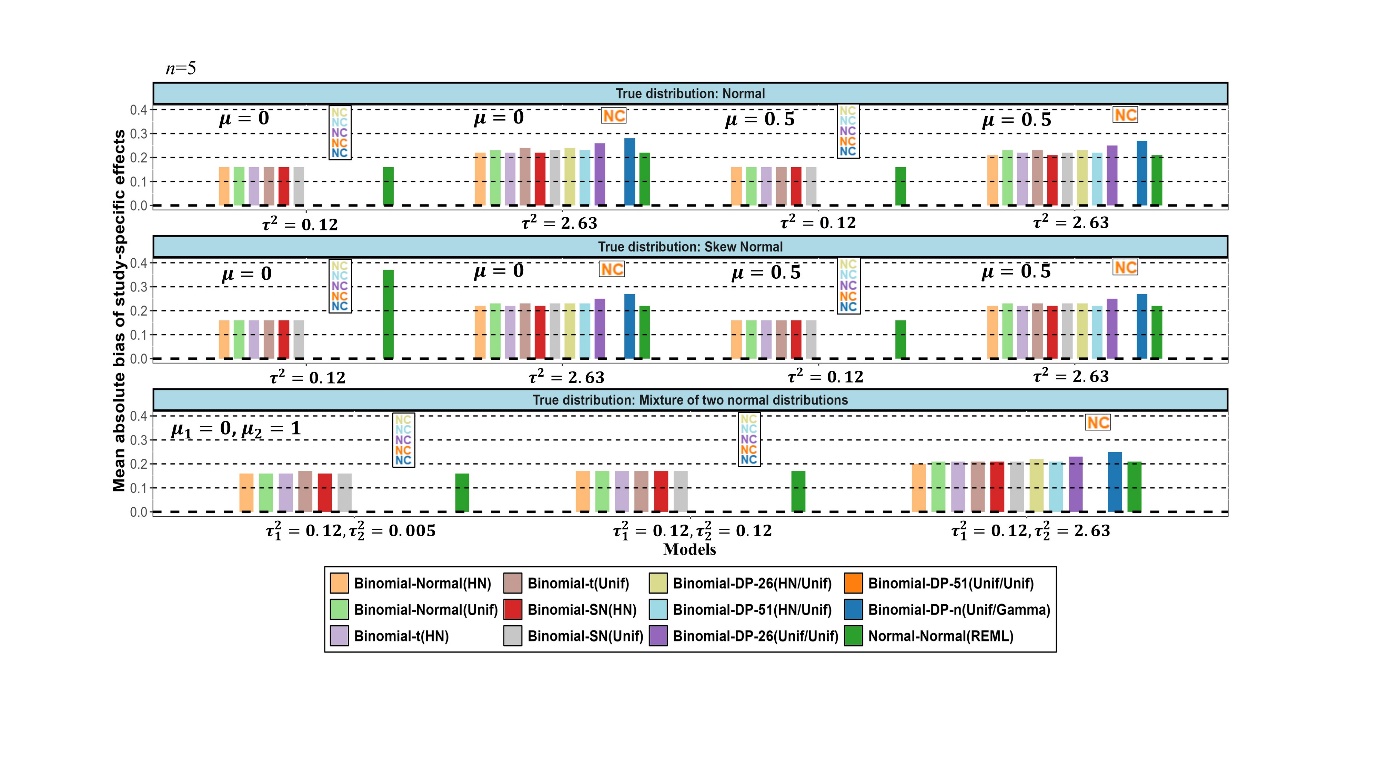
**Supplementary Figure 9.** Simulation results for small meta-analyses (with $n=5$ studies) in terms of mean absolute bias for the study-specific treatment effects averaged within meta-analyses and across meta-analyses. Different $\mu,\mu_{1},\mu_{2},\tau^{2},\tau_{1}^{2}$ and $\tau_{2}^{2}$ values represent the true mean(s) and true variance(s) of the different random-effects distributions used for the data generating process. The names of the models are explained in Table 2. (NC=Non-convergence)


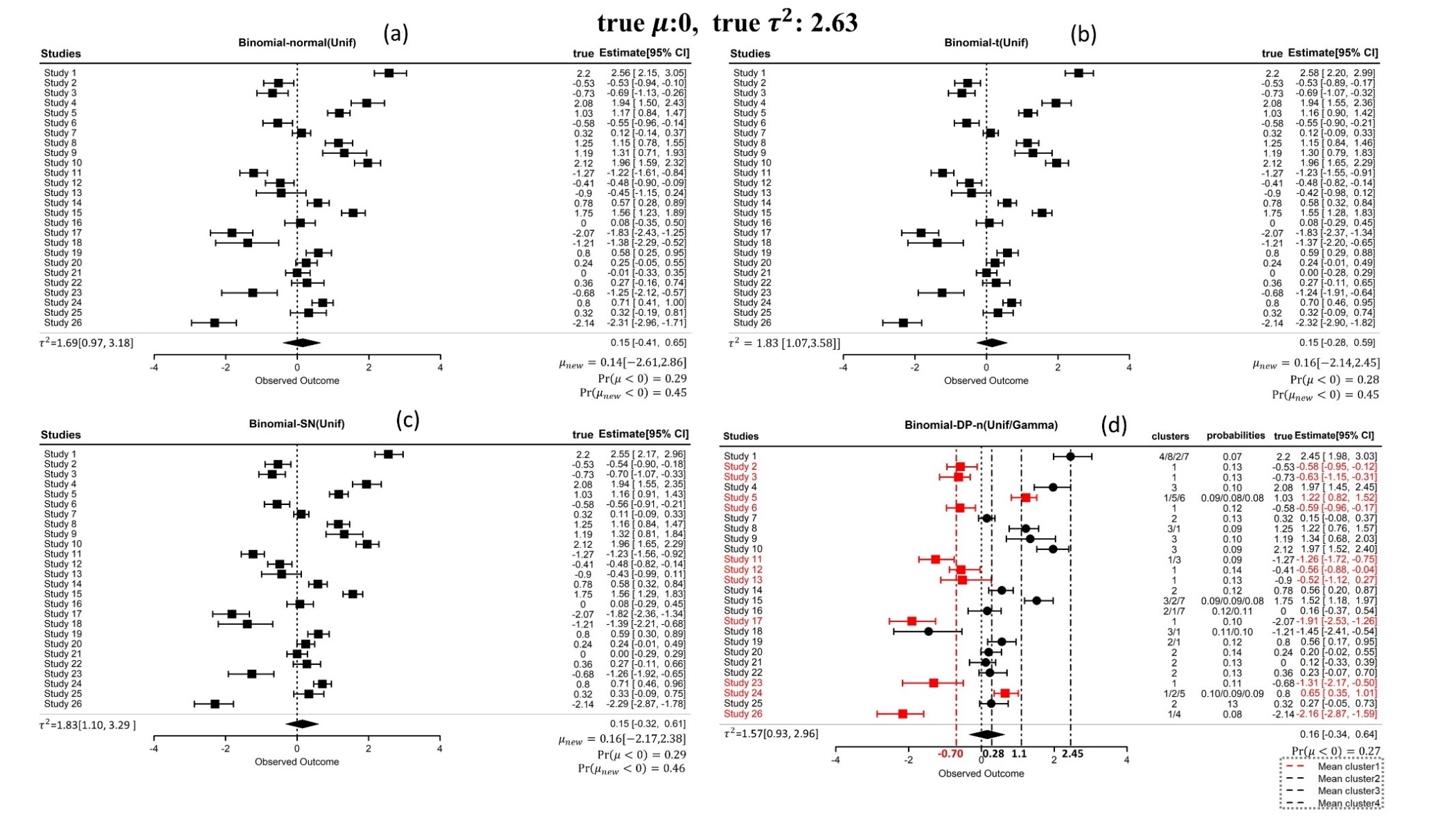


**Supplementary Figure 10.** The estimated study-specific effects for the selected simulated dataset from Scenario 6, with a true mean of 0 and a true random-effects variance of 2.63, are shown using the binomial-Normal(Unif) (panel a), the binomial-t(Unif) (panel b), the binomial-SN(Unif) (panel c), and the binomial-DP-n(Unif/Gamma) (panel d) models. The diamonds represent the estimated mean of the random-effects distribution from each model. The probabilities of the mean of the random-effects distribution or a new study ($\mu_{new}$) to be less than 0 are also presented. In all panels the extra column to the right presents the study-specific true effects. In panel (d), the two extra columns to the right give the cluster assignment and the probability for each study of belonging to the respective cluster. The dashed vertical lines represent the means of the identified clusters. Red studies are those belonging to the dominant cluster and the black ones those belonging to the other clusters.


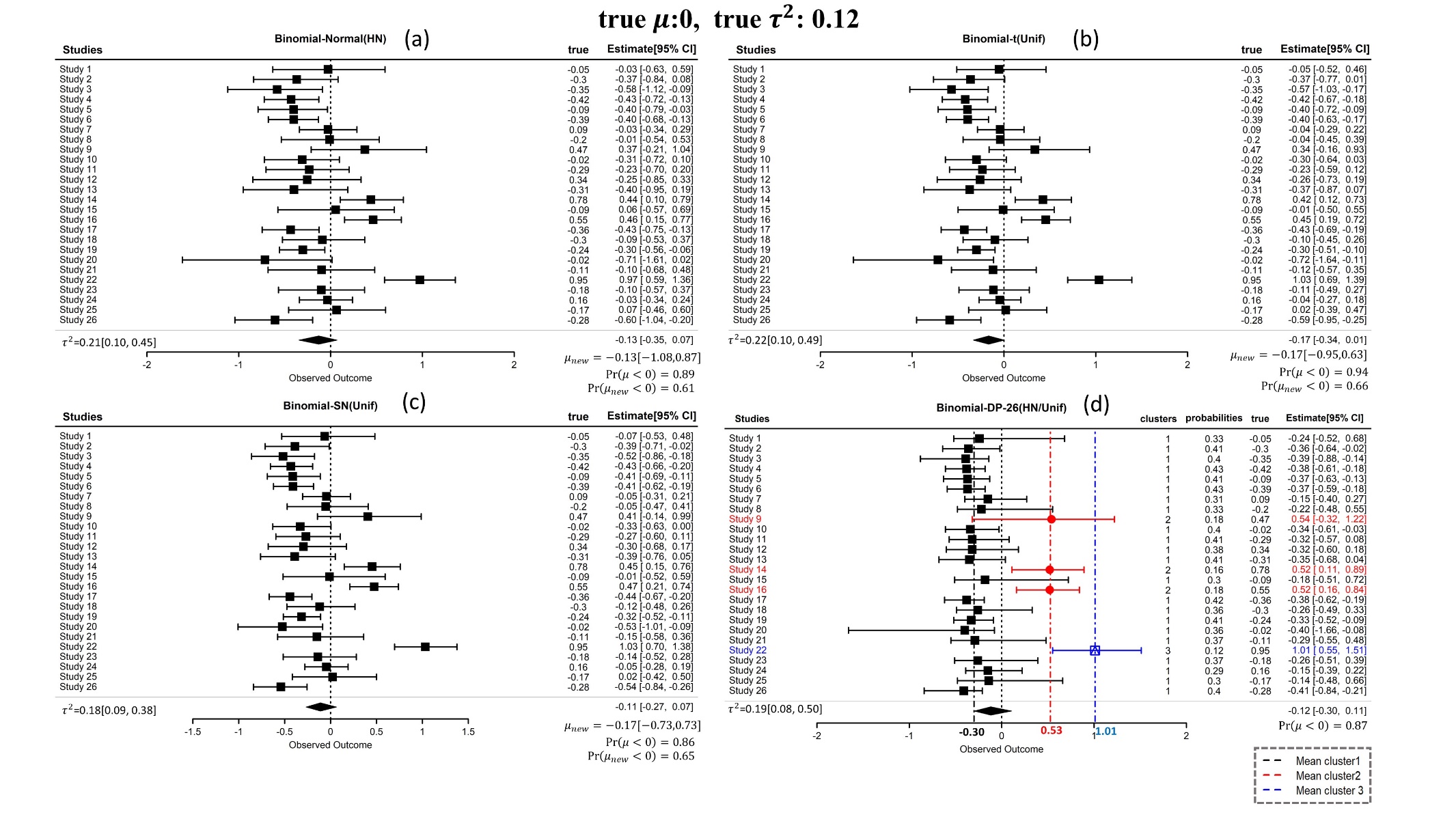


**Supplementary Figure 11.** The estimated study-specific effects for the selected simulated dataset from Scenario 13, with a true mean of 0 and a true random-effects variance of 0.12, are shown using the binomial-Normal(HN) (panel a), the binomial-t(Unif) (panel b), the binomial-SN(Unif) (panel c), and the binomial-DP-26(HN/Unif) (panel d) models. The diamonds represent the estimated mean of the random-effects distribution from each model. The probabilities of the mean of the random-effects distribution or a new study ($\mu_{new}$) to be less than 0 are also presented. In all panels the extra column to the right presents the study-specific true effects. In panel (d), the two extra columns to the right give the cluster assignment and the probability for each study of belonging to the respective cluster. The dashed vertical lines represent the means of the identified clusters. Black, red and blue studies are those belonging to the first, second and third cluster respectively.


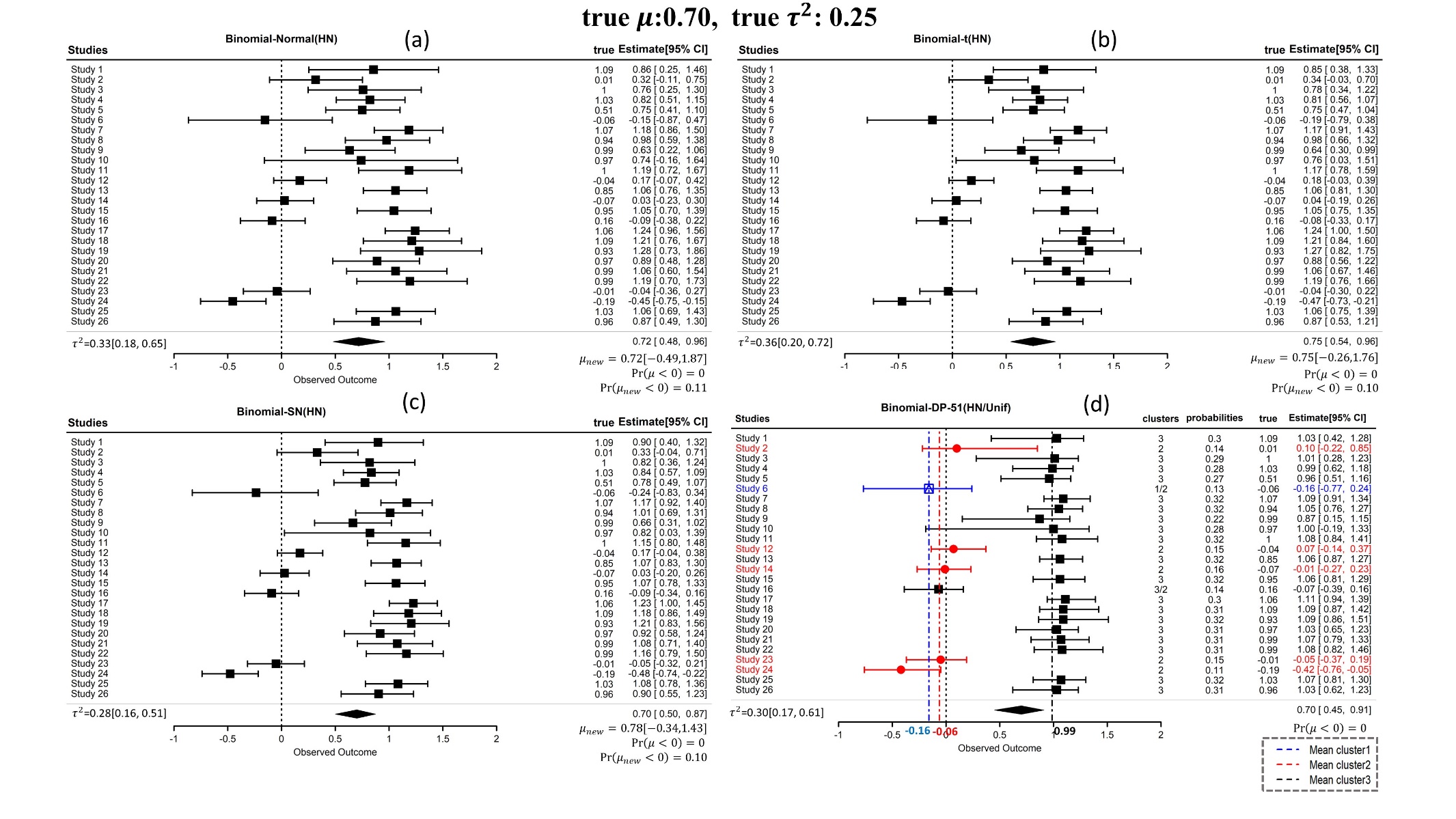


**Supplementary Figure 12.** The estimated study-specific effects for the selected simulated dataset from Scenario 20, with a true mean of 0.70 and a true random-effects variance of 0.25, are shown using the binomial-Normal(HN) (panel a), the binomial-t(HN) (panel b), the binomial-SN(HN) (panel c), and the binomial-DP-51(HN/Unif) (panel d) models. The diamonds represent the estimated mean of the random-effects distribution from each model. The probabilities of the mean of the random-effects distribution or a new study ($\mu_{new}$) to be less than 0 are also presented. In all panels the extra column to the right presents the study-specific true effects. In panel (d), the two extra columns to the right give the cluster assignment and the probability for each study of belonging to the respective cluster. The dashed vertical lines represent the means of the identified clusters. Blue, red and black studies are those belonging to the first, second and third cluster respectively.


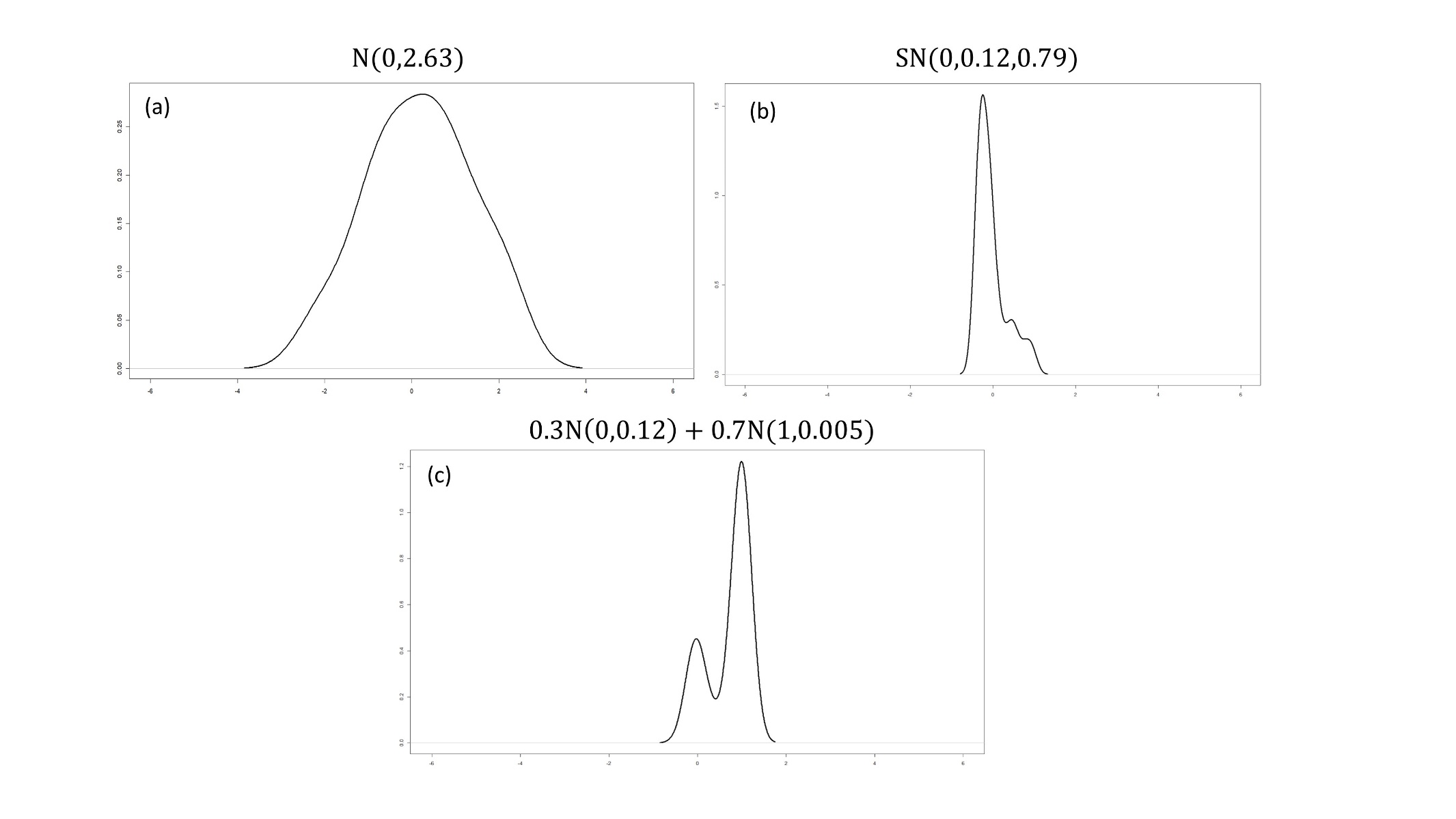


**Supplementary Figure 13**. Distribution of the 26 true study-specific effects of the selected simulated dataset from Scenario 6 (panel (a)), Scenario 13 (panel (b)), and Scenario 20 (panel (c)).


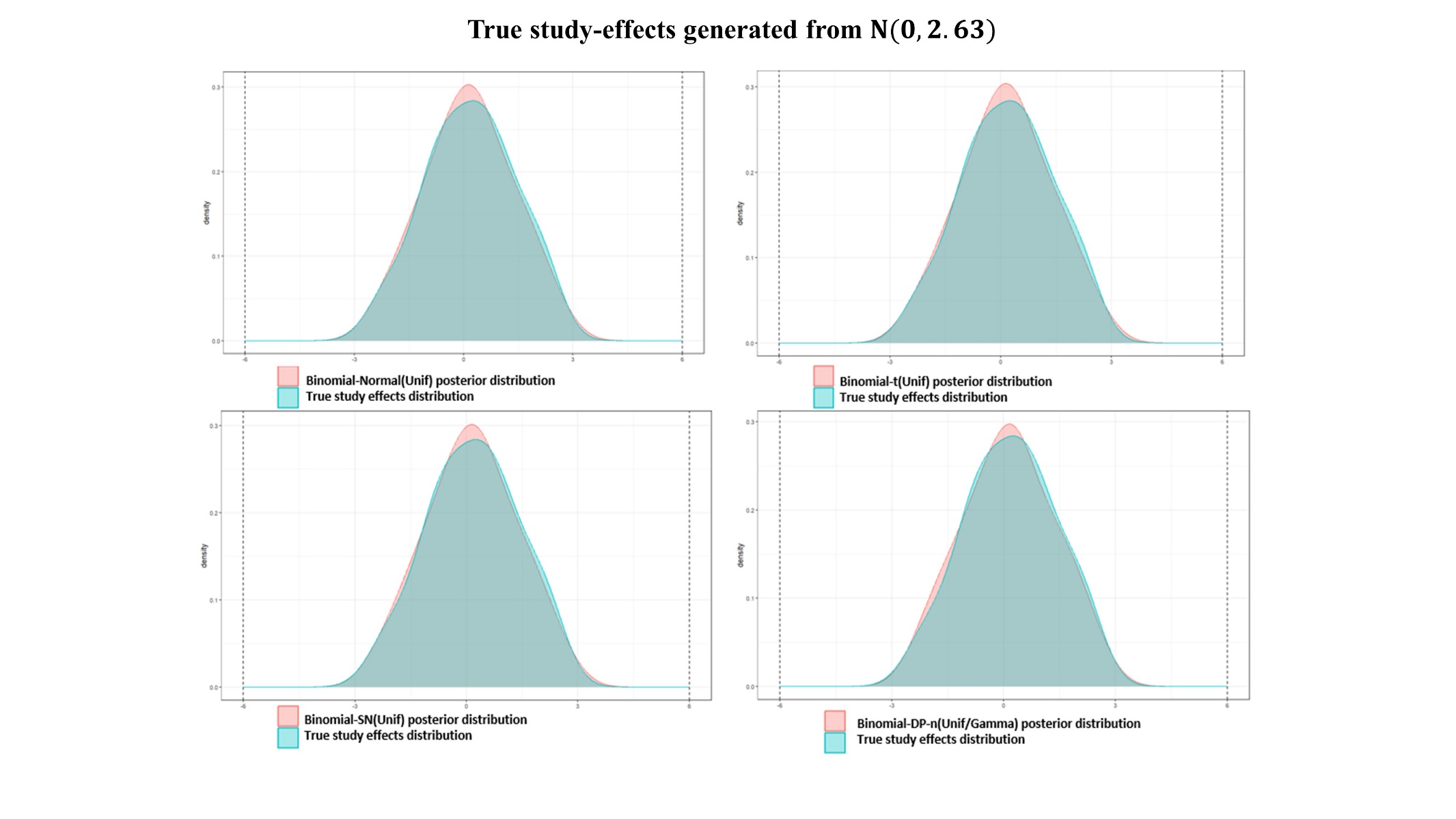


**Supplementary Figure 14**. Overlap between the distribution of the 26 true study-specific effects (Scenario 6) and the posterior distribution of the random effects from each Bayesian model used.


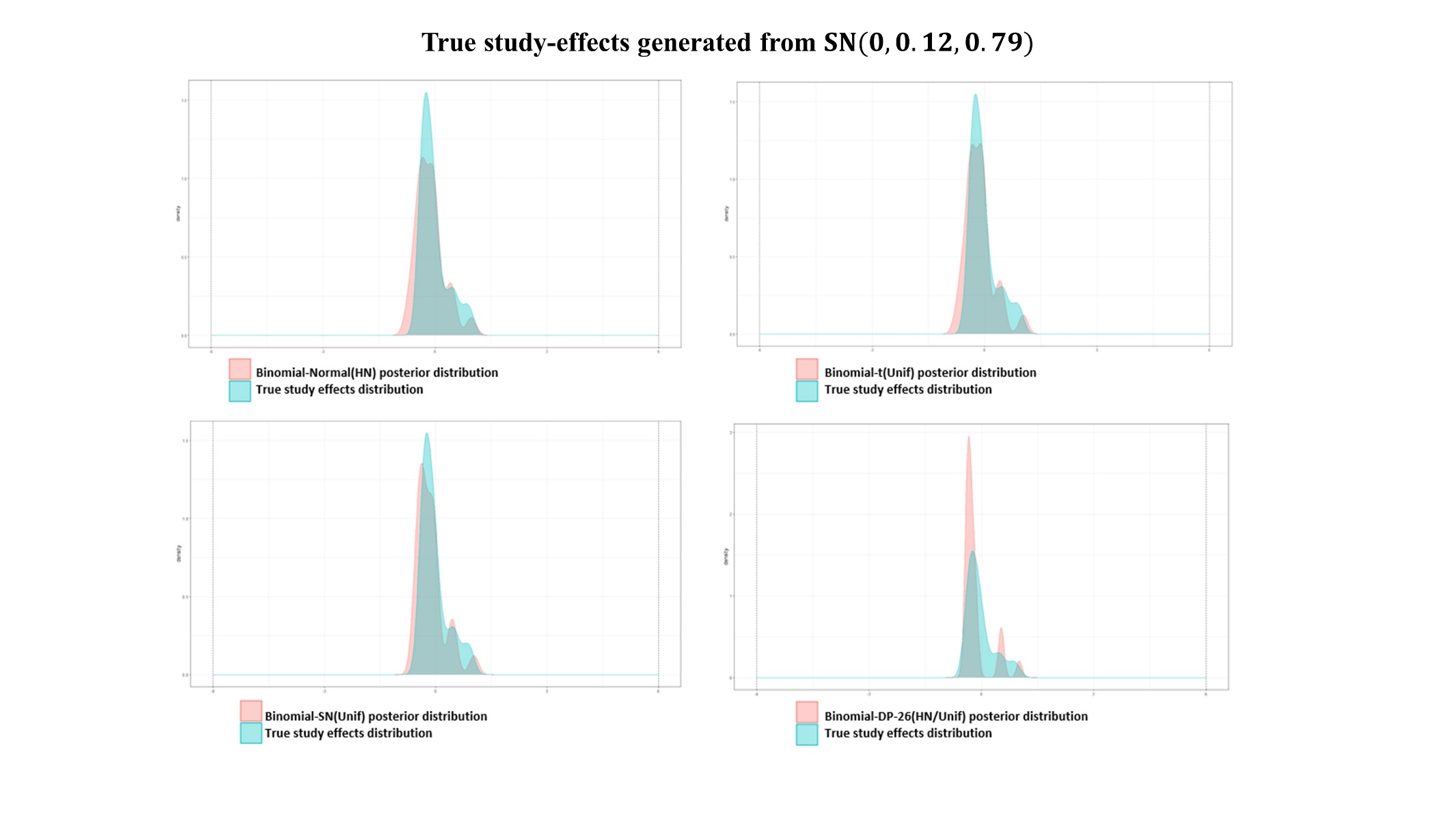


**Supplementary Figure 15.** Overlap between the distribution of the 26 true study-specific effects (Scenario 13) and the posterior distribution of the random effects from each Bayesian model used.


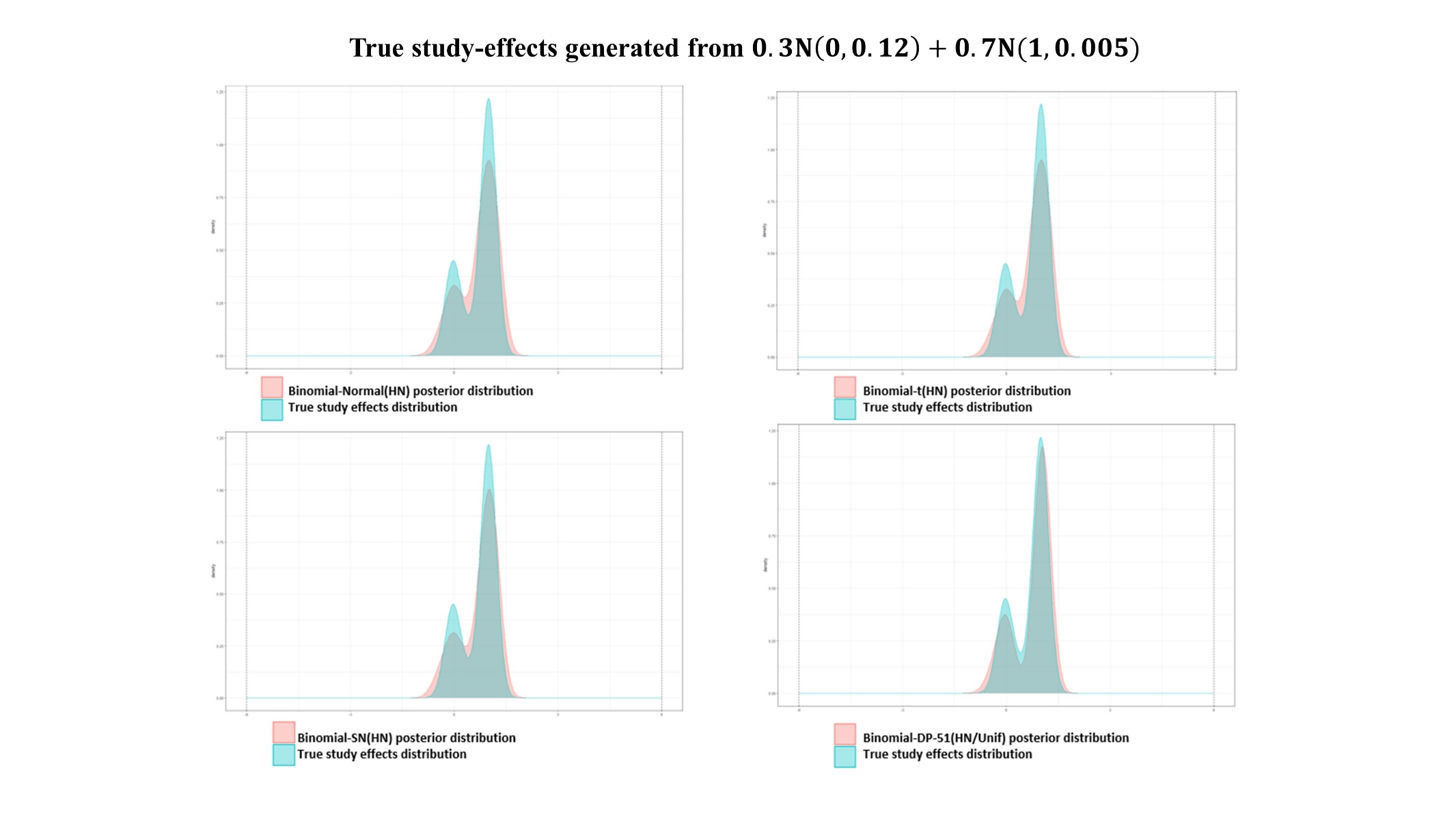


**Supplementary Figure 16**. Overlap between the distribution of the 26 true study-specific effects (Scenario 20) and the posterior distribution of the random effects from each Bayesian model used.

1. Page G. CBnetworkMA: Contrast-Based Bayesian Network Meta Analysis, 2024. URL https://CRAN.R-project.org/package=CBnetworkMA, R package version 0.1.0.

2. Beath KJ. metaplus: An R Package for the Analysis of Robust Meta-Analysis and Meta-Regression. *R J* 2016; 8: 5.

3. Bodnar O, Bodnar T, Thorsén E. BayesMultMeta: Bayesian Multivariate Meta-Analysis, 2022 URL https://CRAN.R-project.org/ package=BayesMultMeta, R package version 0.1.1.

4. Burr D. bspmma: An R Package for Bayesian Semiparametric Models for Meta-Analysis. *J Stat Softw* 2012; 50: 1–23.

5. Hong C, Luo C, Tong G et al. xmeta: A Toolbox for Multivariate Meta-Analysis, 2023. URL https://CRAN.R-project.org/package=xmeta, R package version 1.3.2.

6. Vermunt JK, Magidson J. *LG-Syntax user’s guide: Manual for Latent GOLD 4.5 Syntax module*. Belmont: Statistical Innovations Inc, 2008.

7. Karabatsos G. A menu-driven software package of Bayesian nonparametric (and parametric) mixed models for regression analysis and density estimation. *Behav Res Methods*. 2017; 49: 335-362.

8. Noma H, Nagashima K, Kato S, et al. Meta-analysis Using Flexible Random-effects Distribution Models. *J Epidemiol* 2022; 32: 441–448.

9. Arellano-Valle RB, Azzalini A. The centred parameterization and related quantities of the skew-t distribution. *J Multivar Anal* 2013; 113: 73–90.
